# Emerging insights into the immunosuppressive tumor microenvironment and its implications for glioblastoma immunotherapy

**DOI:** 10.3389/fimmu.2025.1665742

**Published:** 2025-10-24

**Authors:** Niovi Nicolaou, Maria S. Andreou, Christiana M. Neophytou, Panagiotis Papageorgis

**Affiliations:** ^1^ Tumor Microenvironment, Metastasis and Experimental Therapeutics Laboratory, Basic and Translational Cancer Research Center, Department of Life Sciences, European University Cyprus, Nicosia, Cyprus; ^2^ Apoptosis and Cancer Chemoresistance Laboratory, Basic and Translational Cancer Research Center, Department of Life Sciences, European University of Cyprus, Nicosia, Cyprus

**Keywords:** immunotherapy, glioblastoma, immunosuppressive tumor microenvironment, drug resistance, immune checkpoint inhibitors

## Abstract

Glioblastoma is considered the most common and lethal form of brain cancer. Despite tremendous progress in glioblastoma therapeutics, the profound intra- and inter-tumoral heterogeneity of glioblastoma tumors, the difficulty of agents to cross the blood-brain barrier (BBB), the development of drug resistance as well as the immunosuppressive tumor microenvironment (TME) predominantly account for the failure of existing conventional and targeted therapies. Therefore, there is a growing necessity to decipher the complexity of the TME that promotes immunosuppression and to discover innovative strategies targeting both the tumor and its TME to improve patient treatment outcomes. In this comprehensive review, we present the latest evidence implicating various components of the TME in regulating the efficacy of immunotherapies. We also discuss the current challenges and opportunities of immunotherapy in treating glioblastoma, including ongoing clinical trials using immune checkpoints inhibitors (ICIs), CAR-T cell therapy, vaccines, cytokine therapy and oncolytic viruses.

## 1 Introduction

Glioblastoma, formerly known as glioblastoma multiforme (GBM), is defined as a WHO Grade 4 adult-type diffuse glioma, indicating that is a fast-growing tumor (1). The term ‘glioblastoma’ is now used for the *Isocitrate dehydrogenase* (IDH) wildtype tumors only, while the IDH-mutant tumors have been renamed to “Astrocytoma, IDH-mutant, CNS WHO grade 4”. For simplicity, in this review, the term ‘glioblastoma’ will be used throughout for IDH wildtype tumors, unless otherwise specified.

Glioblastoma accounts for around half of all the CNS tumors, making it the most common primary malignancy of the adult brain (2). Although the causes of glioblastoma are not fully understood, evidence suggests that age, obesity, exposure to ionizing radiation and hereditary genetic conditions such as neurofibromatosis (NF), Li-Fraumeni syndrome, tuberous sclerosis (TSC), Turcot Syndrome, and Lynch syndrome, are risk factors for developing the disease (3, 4). The clinical symptoms include cognitive disorder and seizures, slowly progressive impairments of the CNS (motor weakness, sensory and memory loss, visual deficits and speech difficulties), headaches, nausea and vomiting, and changes in personality (5–7). Further to the presentation of these clinical symptoms, glioblastoma is diagnosed through radiological exams, including mostly magnetic resonance imaging (MRI), but also CT (computed tomography) scans, and positron emission tomography (PET) (5, 8–12). Finally, a diagnosis of GMB in adults is made if there is evidence of necrosis, microvascular proliferation, mutation in the telomerase reverse transcriptase (*TERT*) promoter, gene amplification of the epidermal growth factor receptor (*EGFR*), or +7/−10 chromosome copy number changes (1). Classification of glioblastoma is based on histological features, with the three main histological variants being giant cell glioblastoma, gliosarcoma and epithelioid glioblastoma, as well as several histological patterns (1).

Glioblastoma is a highly aggressive and lethal tumor, with a median overall survival of 15–18 months, with only 3% of patients having a progression-free survival (PFS) of more than 5 years (2, 13, 14). Current glioblastoma standard of care therapies include surgery, radiation therapy and temozolomide (TMZ) administration (13). However, TMZ’s efficacy is limited due to systemic toxicity and development of resistance resulting in lack of long-term efficacy and cure (15–17). Therefore, alternative therapeutic strategies are being developed to tackle resistance or improve immunotherapy for glioblastoma. Immunotherapy, especially immune checkpoint blockage (ICB) using ICIs, has revolutionized cancer therapy over the last few years, with exceptional success in several cancer types (18, 19). Unfortunately, initial trials in glioblastoma with ICIs have revealed negative results (20, 21). This therapy failure has mainly been attributed to the highly complex and immunosuppressive glioblastoma TME, which comprises glioma and glioma stem cells (GSCs), immune cells, cells of the nervous system, the brain vascular system, and extracellular matrix (ECM) components (22, 23). Novel ICIs and immunotherapeutic approaches aiming to overcome the challenges posed by the limited glioblastoma immunogenicity are currently in development or being evaluated in clinical trials. This review summarizes the current state of immunotherapy in glioblastoma and discusses the underlying mechanisms by which TME components affect its efficacy or can be exploited for the identification of alternative immunotherapeutic targets in the future.

## 2 Available therapies for glioblastoma

The current standard of care for newly and recurrent diagnosed patients of glioblastoma includes the removal of tumor to the greatest extent that is safe for the patient, followed by radiotherapy with concomitant chemotherapy (13). Other therapeutic options include the alkylating agents Carmustine and Lomustine, the monoclonal antibody Bevacizumab, and Tumor Treating Fields (TTFiels).

### 2.1 Surgery

As recently reviewed (24), several neurosurgical strategies are available, tailored for each patient according to the tumor volume and location. These include resection of the tumor (supramaximal, gross total, subtotal or near-total) and biopsy. Surgery not only helps to minimize tumor volume and improve patients’ overall survival (OS) but also allows surgeons to biopsy the tumor for classification and to design the appropriate radiotherapy regime. Maximal safe resection is recommended as the initial step in treatment since it alleviates symptoms, enhances OS, and boosts the effectiveness of adjuvant therapies.

### 2.2 Radiotherapy

Following surgery, radiotherapy is a cornerstone of glioblastoma treatment and has been shown to increase OS (3). Simple 2D and 3D radiotherapy techniques as well as more modern approaches, such as intensity-modulated radiotherapy (IMRT), can maximize the levels of radiation that reach the tumor site, while minimizing off-targeting to non-cancerous areas of the brain, thus reducing neurotoxicity and other adverse side effects. Following radiotherapy, patients with favorable prognostic factors or methylated O6-methylguanine-DNA methyltransferase (MGMT) promoter, typically receive adjuvant chemotherapy, usually with TMZ as discussed below. This combined approach is often referred to as the Stupp regimen and it is considered the standard of care for newly diagnosed glioblastoma (25).

### 2.3 Approved drugs and implant-based therapy

#### 2.3.1 Temozolomide

TMZ, commercially known as Temodar, has received FDA approval for treating glioblastoma in 2005, based on its improvement in overall OS (26). It belongs to the new class of oral alkylating agents with an imidazole ring, with its chemical designation being 3-methyl-4-oxoimidaz[5,1-d][1,2,3,5]tetrazine-8-carboxamide (27–29). TMZ is a BBB penetrating pro-drug, which gets hydrolyzed under physiological pH to its active drug form, methyltriazen-1yl imidazole-4-carboxamide (MTIC). The drug modifies its targets by adding methyl groups in genomic DNA in guanine and adenine, in N7 and O6, and N3 sites respectively. Methylation causes DNA replication errors and disruption of the mismatch repair (MMR) repair system, which leads to DNA double-strand breaks and eventually programmed cell death. Therefore, the clinical benefit of TMZ is significantly influenced by the methylation of MGMT promoter; patients with methylation on the MGMT promoter show a better response to TMZ. While TMZ is generally well-tolerated, it is accompanied with several side effects including hematological and hepatotoxicity and others (30, 31). Furthermore, glioblastoma has several mechanisms of resistance to TMZ, as reviewed elsewhere (15–17).

#### 2.3.2 Carmustine (BCNU)

BCNU (Bischloroethyl Nitrosourea Carmustine) belongs to the class of N-nitrosoureas [1,3-bis(2-chloroethyl)urea], a monofunctional alkylating agent. It is clinically approved in two forms for glioblastoma treatment. Firstly, intravenous (IV) BCNU was approved in 1977 for treating recurrent glioblastoma, however its use has declined due to the availability of more effective treatments will less toxicity. Secondly, Carmustine Wafers (CWs) marketed as Gliadel^®^, are biodegradable wafers that are implanted in the surgical site during glioblastoma resection, and they release carmustine locally. They were approved by the FDA for recurrent GBM and malignant glioma in 1996 and 2003 respectively (32). Their use is currently limited and remains a controversial topic among neurosurgeons due to the potential side effect of pulmonary fibrosis, and lack of significant evidence on its impact on the quality of life, infections after surgery and possibility of adjuvant therapy (33). In a recent meta-analysis study, Ricciardi et al. (2022), evaluated the OS and progression-free survival (PFS) in newly high-grade glioma patients that received intraoperative implantation of CWs, and concluded that CWs can significantly improve OS, but patients must be carefully selected based on their age and tumor volume to minimize side effects (34).

#### 2.3.3 Lomustine (CCNU)

Lomustine, also known as CCNU (chloroethyl-cyclohexyl-nitrosourea), is a monofunctional alkylating agent of the nitrosourea family, that alkylates DNA and RNA, which triggers cancer cell death through DNA and RNA cross-linking (35). It is lipid-soluble and thus can successfully cross the BBB. It was FDA-approved for the treatment of brain tumors in 1976, and it is still being widely used for recurrent and progressive glioblastoma, administered orally in 6 to 8 weeks intervals (36, 37). Lomustine is given as monotherapy or in combination with procarbazine and vincristine (PCV regime), and it is considered safe with well-controlled side effects (37, 38). CCNU is increasingly considered as the standard of care option for recurrent glioblastoma, as no other treatment has demonstrated superior outcomes in controlled clinical trials (39).

#### 2.3.4 Bevacizumab

Bevacizumab, also known as Avastin^®^, is a human recombinant monoclonal antibody to vascular endothelial growth factor (VEGF), a signal protein central to angiogenesis, that has been used for the treatment of several cancer types, both in monotherapy and in combination therapies (40). In 2009, bevacizumab was FDA-approved for recurrent glioblastoma following two Phase II trials, in which it was given in combination with irinotecan, based on its safety and improvement of quality of life (41, 42). In a recent scoping review (43), PFS benefits, and well-controlled side effects were supported for recurrent glioblastoma from bevacizumab, but no benefits in OS were identified. In fact, the European Medicines Agency (EMA) has rejected the use of bevacizumab for treating recurrent GBM, due to the lack of positive benefit-risk (44). It was suggested that combining bevacizumab with other therapies, like TTFields, might improve therapeutic outcome, but more studies are needed (43).

#### 2.3.5 Optune^®^ device

Optune^®^ device, also known as TTFields, made by NovoCure is a portable wearable device, that was initially approved in 2011 for treating patients with recurrent glioblastoma and in 2015 for newly diagnosed glioblastoma (45). TTFields are alternating electric fields that disrupt cancer cell replication both *in vitro* and *in vivo (* 46). TTFields combined with TMZ have shown significantly improved OS and PFS, however, they have not been adopted as standard care due to several factors such as high cost and inconvenience (47).

## 3 The glioblastoma immunosuppressive tumor microenviroment

**Figure 1:**
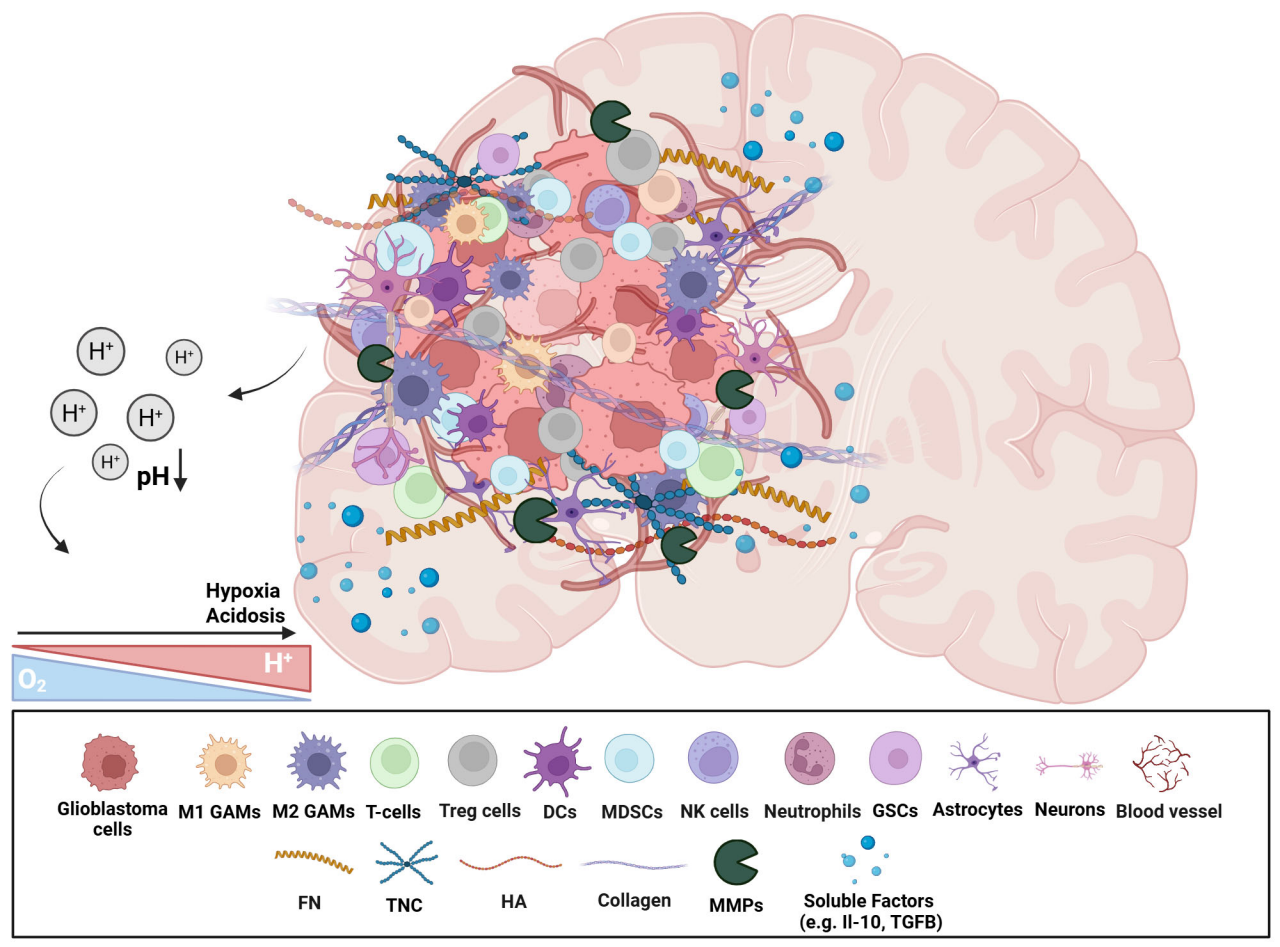
Schematic representation of the immunosuppressive glioblastoma TME. The heterogeneous cellular and non-cellular players of glioblastoma TME are represented. There is an enrichment of immunosuppressive cellular subsets (e.g. Tregs, MDSCs, and GAMs), neural cells, as well as GSCs. Non-cellular aspects include ECM proteins, and soluble factors (e.g. cytokines, growth factors). The hypoxic and acidic environment affects various TME components in numerous ways. This highly complex microenvironment contributes to a strong tumour heterogeneity and immunosuppressive environment, facilitating tumour progression, resistance to therapies, and immune evasion mechanisms. (GAMs, Glioblastoma-associated microglia/macrophages; Tregs, Regulatory T cells; DCs, Dendritic cells; MDSCs, Myeloid-derived suppressor cells; GSCs, Glioblastoma stem cells; FN, Fibronectin; TNC, Tenascin-C; HA, Hyaluronic acid; MMPs, Matrix metalloproteases; ECM, Extracellular matrix). Created in BioRender. Papageorgis, P (2025). https://BioRender.com/qc1ro4l.

The glioblastoma TME is characterized by significant heterogeneity and complexity, encompassing glioma and GSCs, immune and nervous system cells, ECM components and the brain vascular system. Additionally, TME is highly dynamic, characterized by extensive cell-to-cell communication and regulated by factors such as pH and oxygen levels. Glioblastoma lacks infiltration of immune cells, favoring the development of tumorigenic properties, with the immunosuppressive TME playing a crucial role in cancer cell survival and response to therapy (**Figure 1**).

### 3.1 Non-cellular components

#### 3.1.1 ECM

In general, the ECM compromises approximately 20% of the brain mass, and under physiological conditions, it provides structural and biochemical support, as well as regulation of various cellular processes (48). The major components of the ECM, including hyaluronic acid (HA), tenascin-C (TNC), fibronectin (FN), laminin, and collagen, among others, play a vital role in the modulation of invasiveness. In glioblastoma, remodeling, and degrading of the ECM is observed since Matrix metalloproteinases (MMPs), more specifically MMP-2 and MMP-9, are released into the extracellular space. Tenascin-C (TNC), another ECM component, is a matricellular protein (MCP) that is normally expressed at low levels; however, during development and pathological conditions it is highly expressed (49, 50). Glioblastoma cells produce and release TNC and high TNC expression is associated with poor patient survival and disease progression. TNC can interact with multiple proteins (e.g. fibronectin, Toll-like receptors etc.) and thus can promote neovascularization, proliferation, invasiveness, and immunomodulation. Additionally, the presence of TNC stimulates GSCs invasiveness by MMP12 and ADAM metallopeptidase domain 9 (ADAM9) expression and activity, via the c-Jun NH2-terminal kinase pathway. Another ECM component is HA, which activates CD44, a cell surface adhesion protein, stimulating the synthesis and secretion of additional HA, leading to upregulation of MT1-MMP, thus promoting glioblastoma cell infiltration (48). Moreover, FN glycoprotein, also highly expressed in glioblastoma, promotes cell adhesion, differentiation of GSCs, and invasion, and plays a role as a coordinator between ECM and glioblastoma cells (51). Activation of the adhesion kinase/paxillin/Akt signaling pathway is responsible for GSCs adherence and differentiation, while the increase of MMPs’ activity and activation of axis Stat3-ODZ1-RhoA/ROCK, could be responsible for the invasive behavior observed in glioblastoma. Laminin glycoprotein, more specifically laminin-2, -5, and -8, are also found to be highly expressed in glioblastoma patients and they are suspected to have a role in glioblastoma spreading and infiltration (48). Lastly, collagen type I is normally present at low levels in brain tissue, but its expression is slightly elevated in glioblastoma tumors. It is enhanced in the perivascular niche of GSCs, promoting therefore invasiveness via integrin and phosphatidylinositol 3-kinase (PI3K)/Akt signaling pathways.

#### 3.1.2 Physicochemical properties

Physiological factors, such as pH and oxygen concentrations, can affect tumor progression, and immunosuppression (48). The intra-tumoral heterogeneity displayed in glioblastoma alters the nutrient supply and availability of oxygen within the tumor, influencing metabolic properties and energy utilization of cancer cells.

##### 3.1.2.1 Hypoxia

Hypoxic conditions are fundamental drivers of oncogenesis. During hypoxic conditions, glioblastoma cells can adapt and persist due to the high expression of hypoxia-inducible factors (HIFs), a family of transcription factors that are stabilized under low oxygen conditions and are regulated by the inhibition of prolyl-4-hydroxylase 2 (PHD2). This accumulation further activates the expression of downstream targets, including proangiogenic and anti-apoptotic genes. The angiogenesis-induced hypoxia in glioblastoma leads to specific features such as necrotic cores and microvascular hyperplasia, that drive tumor growth and invasion. Additionally, under hypoxia, MMP-2 and MMP-9 expression is increased, and the epithelial-to-mesenchymal transition (EMT) process is induced mainly by transcription factors that have hypoxia response elements (HREs) at their gene promoter regions, such as Twist, Snail, and ZEB. Finally, hypoxia can amplify the activities of immunosuppressive cells, including the influx of M2 macrophages and Tregs at the tumor site (52, 53).

##### 3.1.2.2 Acidosis

The TME as well as intrinsic cellular processes of glioblastoma are affected by acidosis (low pH), which facilitates pro-tumorigenic processes including survival, proliferation, migration, and angiogenesis (52, 53). Tumor acidosis further increases the expression of HIF-1α and HIF-2α and alters the interactions among glioblastoma cells and various TME components, significantly impacting the invasion process (53). It reduces the infiltration and activity of effector lymphocytes and NK cells, further promoting glioblastoma persistence. In addition, drug uptake and efficacy are also affected since acidosis neutralizes radiation-induced reactive oxygen species (ROS) formation, inhibiting apoptosis (52, 53). Thereby, acidosis plays a significant role in fostering a highly immunosuppressive TME.

### 3.2 Cellular components

#### 3.2.1 Immune components

Immune cells found in glioblastoma TME constitute up to 50%; glioblastoma-associated macrophages and microglia (GAMs) are the most prevalent, followed by neutrophils, regulatory T cells (Tregs), dendritic cells (DCs), and myeloid-derived suppressor cells (MDSCs) (54). The immune components present in glioblastoma contribute to the observed immunosuppressive characteristics of the glioblastoma TME.

##### 3.2.1.1 GAMs

GAMs in glioblastoma are composed of macrophages and microglia, which are resident macrophages located in the CNS, both functioning as phagocytic cells. They play a significant and complex role by interacting with glioblastoma cells in various ways that can influence tumor progression (55). While they have the potential to attack glioblastoma cells, they also support tumor growth and invasion. Based on their marker expression and cytokine expression profile, GAMs can be polarized into M1 (anti-tumor) or M2 (pro-tumor) phenotypes. As glioblastoma progresses, however, GAMs tend to be pro-tumorigenic thus favoring an M2 phenotype. The presence of GAMs has been considered to have prognostic value, since higher levels of GAMs are positively correlated with poor prognosis and worsen OS (52). They secrete factors that support tumor growth (e.g. insulin-like growth factor (IGF-1), epidermal growth factor (EGF), platelet-derived growth factor (PDG-F)), as well as anti-inflammatory cytokines (e.g. interleukin-6 (IL-6), IL-10, transforming growth factor beta (TGF-β)), promoting the malignant phenotype of glioblastoma and contributing to the permeability of the BBB (52). More specifically, the proliferation of glioblastoma cells is associated with elevated Ca^2+^ levels in the tumor, which further stimulate ATP-mediated tumor cells that directly interact with GAMs, leading to their activation. In addition, the secretion of IL-6 by GAMs activates the JAK-STAT3 pathway in endothelial cells (ECs) and downregulates intercellular connexins levels, such as connexin43 (Cx43). This contributes to the disruption of the BBB and its increased permeability (56).

##### 3.2.1.2 Neutrophils

In glioblastoma, neutrophils (CD66b^+^ and CD16^+^), a subset of myeloid-derived suppressor cells, upregulate the S100A4 protein, which suppresses the mesenchymal phenotype and facilitates acquired resistance to anti-VEGF therapy (57). They play a role in the oncogenic process of tumor initiation, proliferation, and dissemination via a pro-tumorigenic positive feedback loop. Neutrophils can induce angiogenesis and hinder the functions of DCs, macrophages, and NK cells, thus suppressing the immune system and facilitating in the migration of tumor cells. Neutrophil Extracellular Traps (NETs) secreted by activated neutrophils are extracellular fibrous networks consisting of DNA and proteins (58). Their role in GMB progression is mostly beneficial: they may enhance tumor growth by activating EGFR or TLR4 signaling in tumor cells, they support immune evasion by forming physical barriers for cytotoxic T cells or NK cells and contribute to treatment resistance by activating survival pathways (59).

##### 3.2.1.3 T-cells (CD4^+^, CD8^+^, Tregs)

T lymphocytes, a central component of the adaptive immune system, are essential for anti-tumor immunity. In glioblastoma, however, the anti-tumor response is compromised since CD4^+^/CD8^+^ T-cells constitute only 2% of infiltrating immune cells. Most of these cells show upregulation of inhibitory receptors/immune checkpoints, which signal anergy, exhaustion, and tolerance, thereby promoting further the immunosuppressive nature of glioblastoma (59). In contrast, regulatory T-cells (Tregs) are found to be enriched in glioblastoma infiltrating immune cells. This enrichment promotes the systemic reduction of CD4^+^ T-cells, and the inhibition of the cytotoxic responses of CD8^+^ T-cells, further inducing the effector T-cell anergy and tolerance (60–63). Tregs are suppressor cells, mainly characterized by elevated expression of several transcription factors (e.g. Foxp3, CD25), and cytotoxic T lymphocyte antigen 4 (CTLA-4). Tregs can further inhibit T cell activity by binding to CD80/CD86 on antigen presenting cells (APCs) via CTLA-4. They release immunosuppressive cytokines such as IL-10, IL-4, IL-13, IDO and TGF-β, reducing TNFα and INF-γ levels in effector CD4+ T cells, inhibiting APCs function, and downregulating tumor-specific cytotoxicity within the immune response.

##### 3.2.1.4 NK cells

NK cells (CD56^+^CD3^-^ cells) mediate antigen-independent immune surveillance as effector lymphoid cells (64). Despite their potential for anti-tumor activity, NK cells are found in low levels or impaired within glioblastoma tumors and thus their cytotoxic effects are suppressed by factors such as TGF-β and IL-10 within the TME.

##### 3.2.1.5 DCs

As a diverse class of professional APCs, DCs are central to the activation and regulation of innate and adaptive immunity (61). In normal conditions, DCs are absent from the brain parenchyma, however in pathological conditions such as glioblastoma, DCs can infiltrate brain tissue via afferent lymphatic vessels or endothelial venules. Although DCs have a pivotal role in antitumor immunity, in the TME of glioblastoma, the overexpression of nuclear factor erythroid-related factor (Nef) in DCs results in their suppression and, consequently, a decrease in the effector T cell activation (61).

##### 3.2.1.6 MDSCs

MDSCs are a heterogeneous population of immature myeloid cells that activate immunosuppressive cells and inhibit the release of inflammatory factors thus mediating anti-tumor immunity. In glioblastoma patients, different MDSC populations are present, with the major population being polymorphonuclear CD15^+^CD33^+^HLADR^-^ (PMN-MDSCs) accounting for 82%, followed by lineage-negative (E-MDSCs) at 15% and monocytic (CD14^+^CD33^+^HLADR-; M-MDSCs) at 3% (61, 65). Signal transducer and activator of transcription 3 (STAT3) is a hallmark of MDSCs, and its upregulation regulates MDSCs expansion and tolerogenicity. Factors including IL-10, IL-6, VEGF, GM-CSF, PGE-2, and TGF-β2, found upregulated in glioblastoma, also influence MDSCs expansion. MDSCs are key players in glioblastoma immunosuppressive TME, exerting their effects via various mechanisms including amino acid depletion, oxidative stress, decreased DCs maturation, and the indirect induction of Tregs induced by IL-10 and TGF-β (59). PMN-MDSCs can suppress the antigen-presenting capacity of DCs by upregulating myeloperoxidase (MPO) expression, therefore limiting the ability of DCs to cross-present tumor-associated antigens (TAAs). Additional immunosuppressive activities of MDSCs are regulated by CCAAT/enhancer binding protein β (C/EBPβ) which controls the expression of arginase (ARG1) and inducible nitric oxide synthase (iNOS). These expressions can inhibit T cell growth and migration by interfering with the expression of CD3ζ chain and by inducing the nitration of CCL2 chemokine. iNOS further produces nitric oxide (NO) from amino acid L-arginine, which inhibits the IL-2 signaling pathway in an IFN-γ depended-manner, ultimately impairing T-cell proliferation (59). Moreover, MDSCs suppress NK cell cytotoxicity and cytokine release via ROS production. Crosstalk between GAMs and MDSCs also skews GAMs toward a pro-tumor M2 phenotype.

#### 3.2.2 GSCs

GSCs possess the ability to self-renew and differentiate, contributing to intra-tumoral heterogeneity and playing a key role in tumorigenesis and tumor propagation (48, 54, 66). GSCs activate, regulate, and recruit pro-tumor immune cells. They inhibit T-cell proliferation and cytotoxic T-cell activation while suppressing macrophage-mediated tumor-killing by producing cytokines such as IL-10 and TGFβ. Additionally, GSCs express the TGFβ receptor (TGF-βRII) on their surface, and binding of its ligand triggers the secretion of MMP9 by GSCs. GSCs also interact closely with ECs, creating a perivascular niche, and impacting the glioblastoma progression. Subsequently, GSCs express high levels of proangiogenic growth factors such as VEGF, angiopoietin-1 (Ang-1), bradykinin (BK), IL-8, and stromal cell-derived factor-1 I (SDF-1), which induce their differentiation into ECs and pericytes, further enhancing angiogenesis, migratory abilities, and invasiveness.

#### 3.2.3 Neural components

##### 3.2.3.1 Astrocytes

Astrocytes make up about half of the total volume of the human brain and play a key role in brain physiology and disease, as they are integral components of the BBB. Glioblastoma invasiveness is modulated by astrocytes (48). Glioblastoma cells release EVs into the TME, which are internalized by neighboring astrocytes. Astrocytes then become activated and start secreting elevated amounts of chemokines (e.g. IL-6), enhancing glioblastoma cell invasion and tissue infiltration by increased production MMPs, especially MMP-2 and MMP-9. In addition, glioblastoma invasiveness and migration are modulated by the release of factors, such as glial cell line-derived neurotrophic factor (GNDF) and connective tissue growth factor (CTGF) from astrocytes. GNDF promotes glioblastoma invasion by triggering the activation of rearranged during transfection/GNDF family receptor alpha-1 (RET/GFRa1) receptors and pro-tumoral signaling pathways, such as mitogen-activated protein kinases (MAPK) and PIK3/Akt. Whereas CTGF, when bound to integrin β1, it activates the nuclear factor kappa-light-chain-enhancer of the activated B cells (NF-kB) signaling pathway which secretes additional growth factors, such as TGF-β, further facilitating glioblastoma invasiveness. Moreover, glioblastoma-associated astrocytes upregulate the gap junction protein connexin 43 (Cx43), which facilitates direct communication between astrocytes and glioblastoma cells, promoting further tumor invasion and migration.

##### 3.2.3.2 Neurons

Neurons usually interact indirectly with glioblastoma cells in the TME with different mechanisms, including paracrine stimulation, synaptic transmission, and secretion of neurotransmitters. Glioblastoma proliferation, invasion, and resistance to apoptosis are promoted by the binding of TrkB receptors on glioblastoma cells to molecules released from neurons such as brain-derived neurotrophic factor (BDNF) and neuroligin-3 (NLGN3). Additionally, glioblastoma cells form functional synaptic connections with neurons, enabling electrochemical signaling that influences tumor progression. One example is the AMPA (α-amino-3-hydroxy-5-methyl-4-isoxazolepropionic acid) glutamate receptor, which mediates excitatory postsynaptic potentials, enhancing intracellular calcium signaling, further promoting glioblastoma proliferation, survival, and invasion (67).

##### 3.2.3.3 Oligodendrocytes

Oligodendrocytes are responsible for myelinating axons in the CNS, therefore playing a role in brain physiology, regulating neuronal activities, neural plasticity, and metabolic support (52). In glioblastoma, oligodendrocytes are disrupted leading to worsened tumor-induced damage to neural circuits. They are located at the tumor border niches, suggesting their potential influence in both invasion and recurrence. Oligodendrocytes release cytokines such as angiopoietin-2, which enhances glioblastoma cell motility, and are implicated in promoting angiogenesis contributing to the tumor’s vascular support and sustenance (53).

## 4 Current state of glioblastoma immunotherapy

**Figure 2:**
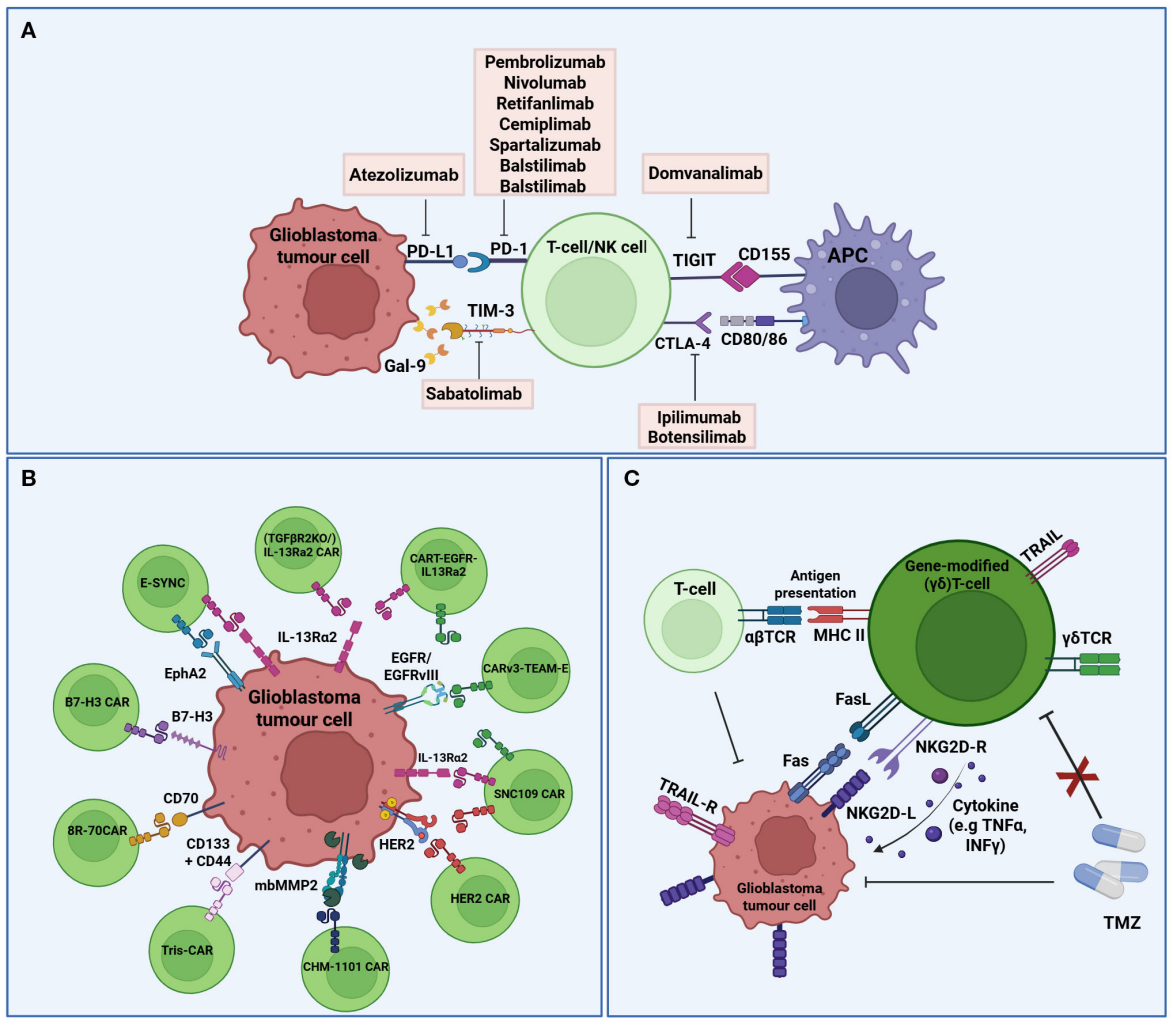
Schematic representation of immune checkpoint inhibitors, CAR-T cells, and (γδ)T-cells in ongoing interventional clinical trials against glioblastoma. **(A)** Immune Checkpoint Inhibitors; Several ICIs and their targets are being investigated in clinical trials to enhance the anti-tumor response. Exhaustion markers (e.g. PD-1, CTLA-4, TIGIT), which are upregulated on the surface of T cells, interact with immune checkpoint molecules (e.g. PD-L1, CD80/86, CD155) expressed on glioblastoma cells and APCs. **(B)** CAR-T cells; Several targetable tumour-associated antigens for glioblastoma CAR T-cell therapy. CAR T-cells are engineered to recognize tumor-associated antigens (e.g. IL13Rα2, HER2, B7-H3, etc) via corresponding CAR constructs, enabling selective tumor cell recognition and cytotoxicity. **(C)** γδT-cells; Gene-modified γδT-cells exhibit direct cytotoxicity against glioblastoma cells and enhance the anti-tumor activity of other immune cells through FasL/TRAIL-mediated apoptosis, antigen presentation, and cytokine secretion. Several antigens and receptors are represented in the same cell for the graphic. (PD1, Programmed cell death protein 1; PD-L1, programmed death-ligand 1; TIM-3, T cell immunoglobulin and mucin domain-containing protein 3; TIGIT, T-cell immunoreceptor with immunoglobulin G1 (Ig1) and immunoreceptor tyrosine-based inhibitory motif (ITIM) domains; CTLA-4, Cytotoxic T-lymphocyte associated protein 4; Gal-9, Galectin-9; MCH, Major histocompatibility complex; APC, Antigen presenting cell; CAR, Chimeric antigen receptor; IL13Rα2, interleukin 13 receptor subunit alpha 2; HER2, human epidermal growth factor receptor 2; mbMMP2, membrane-bound metalloproteinases 2; B7-H3, B7 homolog 3; EphA2, Ephrin type-A receptor 2; EGFR, Epidermal growth factor receptor; TCR, T cell receptor; TMZ, Temozolomide; NKG2D-R, Natural killer group 2 member D receptor; NKG2D-L, NKG2D ligand; TNFα, Tumour necrosis factor 2α; INFγ, Interferon γ; TRAIL, Tumour necrosis factor-related apoptosis-inducing ligand; TRAIL-R, TRAIL receptors). Created in BioRender. Papageorgis, P (2025). https://BioRender.com/mz964iy.

**Figure 3:**
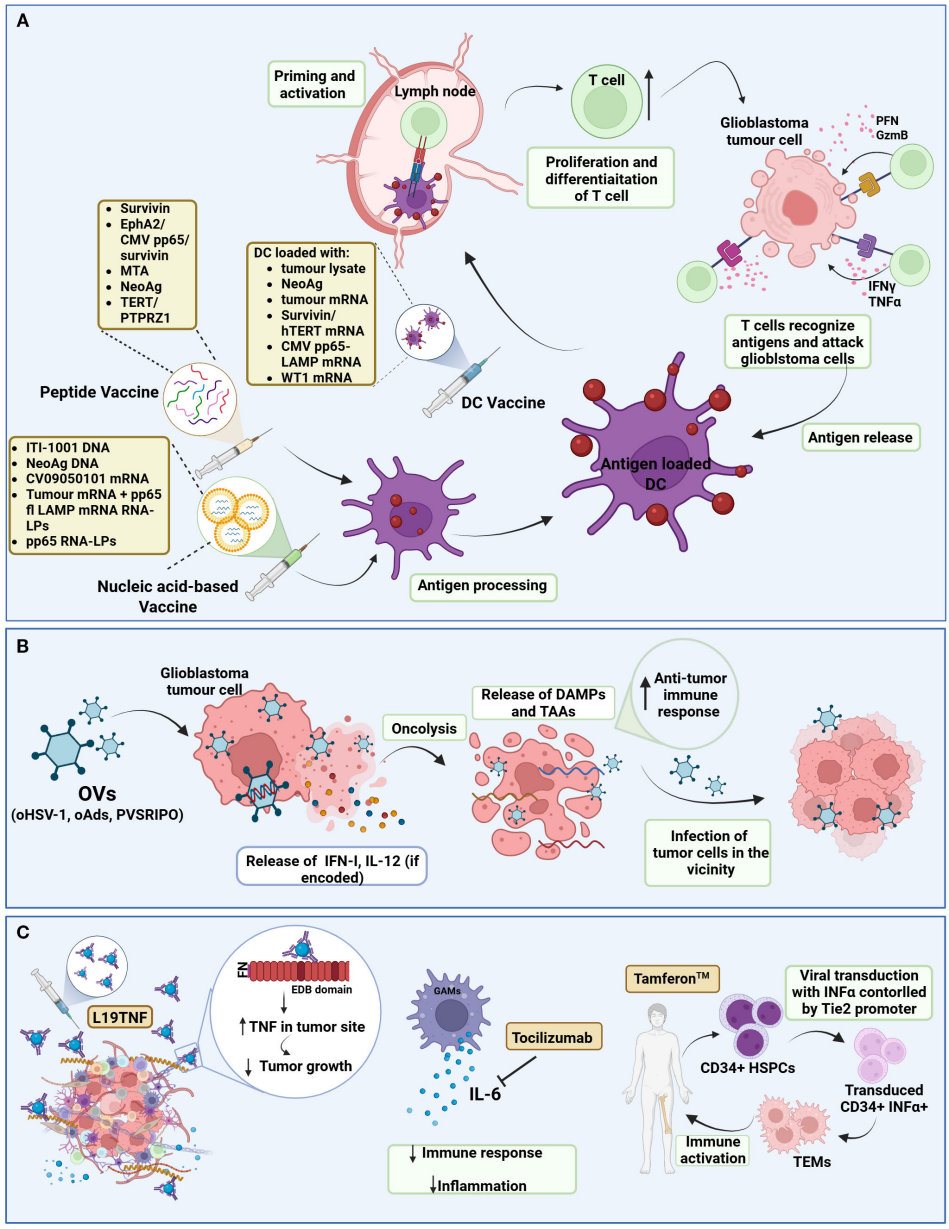
Schematic representation of cancer vaccines, oncolytic viruses, and cytokine therapies in ongoing interventional clinical trials against glioblastoma. **(A)** Cancer Vaccines; Tumour-immune cycle induced by cancer vaccines. After the administration of cancer vaccines (peptide, nucleic acid-based, DC vaccines), the DCs uptake and process the antigens and then present them to MHC II or MHC I via cross-presentation. Antigen-loaded DCs migrate into lymph nodes to prime and activate T-cells. Activated T-cells, proliferate and differentiate into recognizing tumour antigens and targeting glioblastoma cells. Immunogenic dead glioblastoma cells can release additional antigens, leading to the initiation of a subsequent cycle. **(B)** Oncolytic viruses; Genetically engineered OVs selectively replicate within glioblastoma cells while sparing normal cells. This process leads to oncolysis, which not only releases virus progeny to infect neighbouring tumour cells but also exposes DAMPs and TAAs, triggering a robust anti-tumour immune response. **(C)** Cytokine therapies; Cytokine therapies modulate the glioblastoma TME. For example, L19TNF delivers TNF to the TME, reducing tumour growth. Tocilizumab binds to IL-6, decreasing the body’s immune response and inflammation. Tamferon-mediated delivery of INFα via genetically modified CD34+ HSPCs promotes systemic and tumour-localized immune activation. (DC, Dendritic cell; EphA2, Ephrin type-A receptor 2; CMV, Cytomegalovirus; MTA, Mutated-derived tumour antigen; NeoAg, Neoantigen; fl, full length; LPs, lipid particles; TERT, telomerase reverse transcriptase; PTPRZ1, Protein tyrosine phosphatase receptor type Z1; WT1, Wilms; tumour 1; PFN, Perforin; GzmB, Granzyme B; OV, Oncolytic virus; oHSV-1, oncolytic herpes simplex virus-1; oAds, oncolytic adenovirus; INF-I, Interferon Type I; IL-12, Interleukin-12; DAMPs, Damage-associated molecular patterns; TAAs, Tumor-associated antigens; TNF, Tumour-necrosis factor; FN, Fibronectin; EDB, Extra-domain B; GAM, Glioblastoma associated macrophage; IL-6, interleukin 6; TEM, Tie2-expressing macrophages; HSPC, Hematopoietic stem and progenitor cells). Created in BioRender. Papageorgis, P. (2025) https://BioRender.com/n35gcht.

Cancer immunotherapy works by “re-educating” the patient’s immune system to eliminate tumors and therefore holds great promise for cancer therapy (68). The most widely used strategies, often combined, are ICIs, chimeric antigen receptor (CAR) T cells, vaccines and oncolytic viruses. Other immunotherapeutic strategies include other T-cell based therapeutic approaches, cytokine therapy and other targeted immunomodulatory therapies, including monoclonal antibodies. Currently, 104 interventional clinical trials of immunotherapy in glioblastoma have been identified as active (‘recruiting’, “active not recruiting” and “enrolment with invitation”), in ClinicalTrials.gov, accessed on 01/06/2025 (**Tables 1**–**3**, **Figures 2**, **3**).

**Table 1:** ICIs in ongoing interventional clinical trials against glioblastoma - R, “Recruiting”; ANR, “Active, not recruiting”; and EBI, “Enrolling by invitation”.

ICI(s)	Mechanism of action	Phase	Enrolment (estimated or actual)	Status	Study aims	Clinical trial identifier	Ref.
Pembrolizumab (anti-PD-1)	+ Stereotactic radiation + Surgical resection	Ib/II n=10	10	ANR	Assessment of safety/tolerability/feasibility of pembrolizumab and radiation therapy before surgical resection in patients with recurrent glioblastoma	NCT04977375	n/a
	Monotherapy	II	18	ANR	Pharmacodynamic assessment of pembrolizumab in recurrent glioblastoma	NCT02337686	(69)
	Monotherapy	I	60	ANR	Evaluation of early immunologic pharmacodynamics	NCT02852655	(70)
	+ Chemoradiation	IV	36	R	Evaluation of short-term and long-term safety, tolerability and effectiveness of neoadjuvant and adjuvant Pembrolizumab	NCT05235737	n/a
	+ Chemoradiation	II	56	ANR	Exploitation of therapy in newly diagnosed glioblastoma	NCT03899857	n/a
	+ surgery + chemoradiation	II		ANR	Assessment of safety and tolerability in patients with glioblastoma	NCT03197506	n/a
	+ Efineptakin alfa	II	44	R	Efficacy and safety study in recurrent glioblastoma	NCT05465954	(71)
	+ ATL-DC vaccine + poly ICLC	I	40	R	Evaluation of safety, tolerability and efficacy in patients with surgically accessible recurrent/progressive glioblastoma	NCT04201873	n/a
	+ LITT	I/II	34	R	Evaluation of side effects and efficacy in recurrent glioblastoma	NCT03277638	n/a
	+ Optune^®^ + TMZ	II	40	ANR	Assessment of safety, efficacy and effect on PFS of the combinational treatment in newly diagnosed glioblastoma	NCT03405792	(72)
	+ Optune ^®^ + TMZ	III	741	ANR	Evaluation of overall survival in newly diagnosed glioblastoma	NCT06556563	n/a
	+ Optune ^®^ + MLA	II	20	R	Evaluation of safety and feasibility in patients with recurrent or progressive glioblastoma	NCT06558214	n/a
	+/- (Opalarib + TMZ)	II	78	R	Evaluation of safety and efficacy of combinational treatment in patients with recurrent glioblastoma at their first or second relapse	NCT05463848	n/a
	+ M032 (oHSV)	I/II	28	R	Assessment of safety and tolerability in recurrent GBM	NCT05084430	n/a
	+ Allogeneic CMV-specific T cells	I/II	58	R	Assessment of maximum tolerated doses of combination therapy in newly diagnosed GBM	NCT06157541	n/a
Nivolumab (anti-PD-1)	Monotherapy	II	61	ANR	Testing the efficacy of nivolumab in patients with IDH-mutant gliomas with and without hypermutator phenotype	NCT03718767	(73)
	Monotherapy	I	20	R	Evaluation of side effects and improvements in quality of life of nivolumab administered before and after surgery in treating children and young adults with recurrent high-grade gliomas	NCT04323046	n/a
	+ TMZ	II	103	ANR	Assessment of benefits of giving nivolumab in together with TMZ versus TMZ alone on OS in newly diagnosed elderly GMB patients	NCT04195139	(74)
	+ Crizanlizumab	I/II	33	R	Evaluation of the efficacy, safety and tolerance monotherapy and combination therapy in patients with advanced glioblastoma newly diagnosed unmethylated glioblastoma	NCT05909618	n/a
	+ Bevacizumab (anti-VEGF)	II	90	ANR	Evaluation of safety, tolerability and efficacy of nivolumab when given in combination with low doses of bevacizumab	NCT03452579	(75)
	+ RT + Bevacizumab	II	39	ANR	Assessment of effectiveness of nivolumab added to the radio/anti-VEGF therapy in recurrent MGMT methylated glioblastoma	NCT03743662	n/a
	+ BMS-986205 (IDO inhibitor) + RT +/- TMZ	I	18	ANR	Determination of the safety and tolerability of combinational treatment in newly diagnosed MGMT promoter methylated and unmethylated glioblastoma.	NCT04047706	(76)
	+ ipilimumab + TMZ	II	47	ANR	Assessment of overall survival in patients with newly diagnosed glioblastoma or gliosarcoma	NCT04817254	n/a
Retifanlimab (anti-PD-1)	+ anti-GITR + SRS	II	39	ANR	Safety, immunogenicity, and therapeutic efficacy in recurrent glioblastoma	NCT04225039	(77)
Retifanlimab (anti-PD-1)	+ RT + Bevacizumab +/- Epacadostat (IDO-1 inhibitor)	II	51	ANR	Safety and efficacy assessment of combinational treatment in recurrent glioblastoma	NCT03532295	(78)
	+ Personalized neoantigen DNA vaccine	I	12	R	Assessment of safety and immunogenicity in newly diagnosed, MGMT promoter unmethylated glioblastoma	NCT05743595	n/a
Cemiplimab (anti-PD-1)	+ INO-5401 + INO-9012 + RT + TMZ	I/II	52	ANR	Evaluation of treatment safety, immunogenicity and preliminary efficacy in newly diagnosed GBM patients	NCT03491683	(79)
	+ ASP8374 (Anti-TIGIT)	Ib	14	ANR	Evaluation of safety and efficacy in recurrent malignant glioma	NCT04826393	n/a
Balstilimab (anti-PD-1) + Botensilimab (anti-CTLA-4)	+ DOX + Sonocloud-9 device (SC-9)	IIa	25	R	Establishment of safety and feasibility of delivering immune modulating drugs in this manner, and evaluation of treatment efficacy	NCT05864534	(80)
Atezolizumab (anti-PD-L1)	+TMZ +/- RT	I/II	80	ANR	Evaluation of combining atezolizumab with standard of care in newly diagnosed glioblastoma	NCT03174197	(81)
	monotherapy	II	80	R	Assessment of therapeutic benefit of neoadjuvant atezolizumab in patients with recurrent low mutational burden glioblastoma	NCT06069726	n/a
	+ D2C7-IT (anti- EGFRwt/EGFRvIII)	I	18	ANR	Evaluation of combinational treatment in recurrent glioblastoma	NCT04160494	n/a
	+ FSRT radiation	I	12	R	Evaluation of immunogenic effect in newly diagnosed glioblastoma	NCT05423210	n/a
	+ Nivolumab + RT	II/III	159	ANR	Assessment of combination therapy on progression-free survival compared to standard of care in newly diagnosed MGMT unmethylated glioblastoma	NCT04396860	(82)
	+ Nivolumab + Surgical removal	I	63	ANR	Assessment of safety and effectiveness of combinational treatment in recurrent glioblastoma	NCT04606316	(82)
	Cabozantinib (inhibition of tyrosine kinases involved in angiogenesis, motility & invasion)	I/II	6	R	Assessment of safety and efficacy of combinational therapy in recurrent glioblastoma	NCT05039281	n/a
	+ Ipatasertib (Akt inhibitor)	I/II	87	ANR	Assessment of safety and MTD	NCT03673787	(83)
Ipilimumab (anti-CTLA-4)	+ Nivolumab	I	18	R	Determination of the safety and feasibility of the proposed investigational (neo-) adjuvant treatment regimen in patients with resectable recurrent glioblastoma	NCT06097975	(84)
Sabatolimab (anti-TIM-3)	+ Spartalizumab (anti-PD-1)	I	16	ANR	Assessment of safety of MBG453 given in combination with spartalizumab and SR in patients with recurrent GBM	NCT03961971	n/a
Domvanalimab (anti-TIGIT)	+ Zimberelimab (anti-PD-1)	0/I	46	ANR	Exploratory study of combination therapies in recurrent glioblastoma	NCT04656535	n/a

PD1, Programmed cell death protein 1; TIM-3, T cell immunoglobulin and mucin domain-containing protein 3; GITR, Glucocorticoid-Induced Tumor Necrosis Factor-related protein; TIGIT, T-cell immunoreceptor with immunoglobulin G1 (Ig1) and immunoreceptor tyrosine-based inhibitory motif (ITIM) domains; CTLA-4, Cytotoxic T-lymphocyte associated protein 4; TMZ, Temozolomide; PD-L1, programmed death-ligand 1; LITT, Laser Interstitial Thermotherapy; RT, Radiotherapy; oHSV, oncolytic Herpes simplex virus; SRS, stereotactic radiosurgery; ATL-DC, autologous tumor lysate pulsed dendritic cell; poly ICLC, Polyinosinic-Polycytidylic acid; MLA, MRI-guided laser ablation; IDO, indoleamine 2, 3-dioxygenase 1; CMV, Cytomegalovirus; FSRT, fractionated stereotactic radiotherapy; n/a, not applicable.

**Table 2:** T-cell based therapies in ongoing interventional clinical trials against glioblastoma – R, “Recruiting”; ANR, “Active, not recruiting”; and EBI, “Enrolling by invitation”.

CAR-T cells							
Target (s)	Mechanism of action	Enrolment (estimated or actual)	Phase	Status	Study aims	Clinical trial identifier	Ref.
CD133 + CD44	Inverse correlated dual-target, truncated IL7Ra modified CAR -expressing autologous T-lymphocytes (**Tris-CAR-T cells**)	10	I	R	Evaluation of safety, distribution, tumor progression, and changes in target expression and tumor biology over time.	NCT05577091	(85)
CD70	Ex-Vivo expanded autologous IL-8 receptor (CXCR2) modified CD70 CAR (**8R-70CAR**) T cells	39	I	R	Assessment of safety and feasibility in newly diagnosed CD70 positive adult GBM patients who have undergone surgery	NCT05353530	(86)
	Ex-Vivo expanded autologous IL-8 receptor (CXCR2) modified CD70 CAR (8R-70CAR) T cells	18	I	R	Assessment of safety and feasibility in CD70+ Adult GBM and Pediatric High-Grade Gliomas (pHGG)	NCT06946680	n/a
EphA2 + IL-13Rα2	***E-SYNC T cells*** (Autologous Anti-EGFRvIII synNotch Receptor Induced Anti-EphA2/IL-13Ra2 CAR T Cells) +	20	I	R	Evaluation of safety, side effects, and best dose after lymphodepleting chemotherapy in treating patients with EGFRvIII + glioblastoma.	NCT06186401	n/a
EGFRvIII + EGFR	**CARv3-TEAM-E T Cells** (Autologous T lymphocytes)	21	I	R	Evaluation of safety and dose of in newly diagnosed and recurrent glioblastoma	NCT05660369	(87)
IL-13Rα2	IL13Ra2-specific CAR Tcm cells (**IL13Ra2-CAR/CD19t+ Tcm**)	65	I	ANR	Assessment of feasibility & safety in recurrent gliomas	NCT02208362	(88)
		10	I	R	Safety and feasibility assessment	NCT04661384	n/a
	IL13Ra2-specific-hinge-optimized-4-1BB-CAR/truncated CD19-expressing Autologous TN/MEM Lymphocytes +/- Nivolumab +/- ipilimumab	60	I	R	Assessment of safety and feasibility in recurrent or refractory glioblastoma	NCT04003649	n/a
	IL13Ra2-targeting CAR-T cells with TGFβR2 Knockout (**TGFβR2KO/IL13Rα2 CAR T cells**)	27	I	R	Assessment of safety and side effects in recurrent or progressive glioblastoma or IDH-mutant grade 3 or 4 astrocytoma	NCT06815029	n/a
EGFR + IL-13Rα2	Autologous T cells transduced with a bicistronic lentiviral vector containing a murine scFv targeting EGFR and a humanized scFv targeting IL13Ra2 (**CART-EGFR-IL13Ra2 Cells**)	66	I	R	Safety and feasibility evaluation in patients with EGFR-amplified recurrent glioblastoma	NCT05168423	(89, 90)
HER2	HER2(EQ)BBζ/CD19t+ T cells	29	I	ANR	Assessment of safety and dose in recurrent or non-responsive glioblastoma	NCT03389230	n/a
EGFR, EGFRvIII, HER2 + IL-13Rα2	**SNC109 CAR-T cells**	50	I	EBI	Evaluation of safety, tolerance, and pharmacokinetics of SNC109 in patients with recurrent glioblastoma	NCT06616727	n/a
	B7-H3-targeting CAR-T	30	I	ANR	Evaluation of safety, tolerability, effectiveness and MTD for phase II in recurrent glioblastoma	NCT05241392	n/a
		48	I	R	Evaluation of efficacy of locoregional delivery of B7-H3	NCT05835687	(91)
		52	I	R	Evaluation of safety, efficacy and MTD for phase II for progressive grade 4 glioma	NCT06482905	n/a
		36	I	R	Safety evaluation in recurrent or refractory glioblastoma	NCT05366179	n/a
		39	I	R	Assessment of manufacturing feasibility and safety of locoregional administration of B7-H3CART into the CNS of adults with recurrent glioblastoma	NCT05474378	(92)
Membrane-bound MMP-2	**CHM-1101 CAR-T** (Chlorotoxin-CD28-CD3z-CD19t-expressing CAR T-cells)	42	Ib	ANR	Evaluation of safety, best dose and effectiveness in MMP2+ recurrent, aggressive and progressive glioblastoma	NCT05627323	(93)
		19	I	R	Evaluation of safety, best dose and effectiveness in MMP2+ recurrent, aggressive and progressive glioblastoma	NCT04214392	(94)
(γδ)T- cells							
Mechanism of action			Phase	Status	Study aims	Clinical trial identifier	
Gene-modified (γδ)T-cells + TMZ		22	I	ANR	Evaluation of safety and tolerability in newly diagnosed glioblastoma	NCT04165941	(95)
Activated, gene-modified allogeneic or autologous (γδ)-T cells (DeltEx) + maintenance TMZ		4	Ib/II	ANR	Determination of safety, tolerability and ability to delay recurrency in newly diagnosed or recurrent glioblastoma	NCT05664243	n/a

CAR, Chimeric antigen receptor; CD, cluster of differentiation; IL7Rα, Interleukin receptor alpha; IL-8, Interleukin 8; EGFRvIII, endothelial growth factor receptor variant III; EGFR, Epidermal growth factor receptor; HER2, human epidermal growth factor receptor 2; IL-13Rα2, Interleukin 13; MMP-2, matrix metalloproteinase-2; MTD, maximum tolerated dose; CNS, central nervous system; EphA2, ephrin type-A receptor 2; scFV, single=chain variable fragment; B7-H3, B7 homolog 3; TMZ, temozolomide; Tcm, central memory T cells; TN/MEM, naive and memory T cells; n/a, not applicable.

**Table 3:** Vaccines, oncolytic viruses, cytokine therapy and other targeted immunotherapies in ongoing interventional clinical trials against glioblastoma - R, “Recruiting”; ANR, “Active, not recruiting”; and EBI, “Enrolling by invitation”.

Type of immunotherapy		Target (s)/mechanism of action	Phase	Enrolment (estimated or actual)	Status	Study aims	Clinical trial identifier	Ref.
Peptide vaccines		Survivin peptide vaccine (SurVaxM) + TMZ	II	66	ANR	Evaluation of side effects in newly diagnosed glioblastoma patients	NCT02455557	(96)
		SurVaxM + TMZ	II	247	ANR	Determination of whether adding SurVaxM to standard of care TMZ chemotherapy is better than TMZ treatment alone for patients with newly diagnosed glioblastoma	NCT05163080	(97)
		MTA-based vaccine + TTFs + chemoradiation	I	13	ANR	Assessment of safety & tolerability in glioblastoma	NCT03223103	(98)
		TERT/PTPRZ1 peptide vaccine (with synthetic melanin + TLR9 agonist) + radio chemotherapy	I/IIa	35	R	Assessment of safety and immunological efficacy in newly diagnosed glioblastoma patients	NCT06622434	n/a
		Personalized NeoAg + poly-ICLC (NeoVax) + RT +/- pembrolizumab +/- TMZ	I	56	R	Safety evaluation of the personalized vaccine in newly diagnosed GBM	NCT02287428	(99)
		P30-linked EphA2, CMV pp65, and survivin vaccine (ETAPA I)	Ib	24	ANR	Safety evaluation in newly diagnosed, unmethylated & untreated glioblastoma patients	NCT05283109	n/a
DC vaccines		DC loaded with autologous tumor lysate	II	136	ANR	Assessment of overall survival in newly diagnosed glioblastoma patients with confirmed gross-total resection	NCT03395587	(100)
		DC vaccine loaded with autologous tumor lysate + TMZ	II	28	R	Assessment of safety and progression-free survival in glioblastoma patients after surgery and standard radiochemotherapy treatment	NCT04523688	n/a
		DCs loaded with multiple tumor NeoAg peptides + TMZ	I	11	ANR	Determination of safety & preliminary efficacy in adult postoperative newly diagnosed glioblastoma patients	NCT04968366	(101)
		DCs loaded with multiple tumor neoantigen peptides	I	12	R	Evaluation of safety, tolerability and efficacy in recurrent glioblastoma	NCT06253234	n/a
		DOC1021 + pINF + SOC	II	180	R	Evaluation of the safety of DOC1021 + pINF and survival when treated alongside with SOC in newly diagnosed glioblastoma patients (IDH-wt)	NCT06805305	n/a
		DCs loaded with Survivin/hTERT mRNA derived from autologous GSCs	II/III	60	R	Comparison of DC therapy against cancer stem cells and standard therapy in primary treated patients with IDH wild-type, MGMT-promotor methylated glioblastoma	NCT03548571	n/a
		DCs loaded with CMV pp65-LAMP mRNA + chemoradiation +-varlilumab	II	43	ANR	Evaluation of safety, feasibility, and immune response efficacy in patients with newly diagnosed glioblastoma	NCT03688178	n/a
		DCs loaded with autologous tumor lysate	I/II	76	R	Evaluation of treatment tolerability, efficacy and effect on the immune response & identification of possible correlation between methylation status of MGMT and tumor response.	NCT04801147	n/a
		DC vaccine loaded with WT1 mRNA + TMZ	I/II	20	ANR	Determination of overall and progression-free survival of patients with newly diagnosed glioblastoma	NCT02649582	(102)
Nucleic acid-based vaccines		ITI-1001 DNA vaccine + maintenance TMZ	I	10	ANR	Evaluation of the safety, tolerability, immunogenicity, and preliminary efficacy in newly diagnosed GBM	NCT05698199	n/a
		Personalized neoantigen DNA vaccine + INO-9012	I	9	ANR	Assessment of safety, feasibility, and immunogenicity in newly diagnosed, unmethylated glioblastoma patients	NCT04015700	n/a
		CV09050101 mRNA vaccine	I	37	ANR	Evaluation of safety and tolerability in patients with surgically resected unmethylated glioblastoma or astrocytoma	NCT05938387	(103)
		Autologous total tumor mRNA and pp65 fl LAMP mRNA loaded DOTAP liposome vaccine (RNA-LPs)	I/II	28	R	Demonstration of manufacturing feasibility and safety, and determination of the MTD in adult patients with newly diagnosed GBM (MGMT unmethylated)	NCT04573140	n/a
		Pp65 RNA-LPs	I	37	R	Investigation of MTD in recurrent glioblastoma	NCT06389591	n/a
Oncolytic viruses	**oHSV-1**	M032 (express IL-12)	I	29	ANR	Determination of safety, tolerability and MTD in glioblastoma patients not eligible for surgical resection	NCT02062827	(104)
		rQNestin (express ICP4) +/- cyclophosphamide	I	62	R	Safety assessment in recurrent glioma	NCT03152318	(105)
		G207	II	35	R	Assessment of efficacy and safety in recurrent/progressive pediatric high-grade gliomas	NCT04482933	n/a
		G207	I	24	R	Safety evaluation in children with recurrent or refractory cerebellar brain tumors	NCT03911388	(106)
		C134	Ib	12	EBI	Safety & tolerability evaluation of re-dosing of virus in recurrent malignant glioma	NCT06193174	n/a
		C134	Ib	19	ANR	Safety assessment in recurrent resectable glioblastoma	NCT03657576	n/a
	**Adenovirus**	DNX-2401	I	36	R	Determination of MTD in recurrent high-grade gliomas	NCT03896568	n/a
		NSC-CRAd-S-pk7	I	36	R	Study of the effect of multiple repeated doses in recurrent high-grade gliomas	NCT05139056	(107)
		NRG103	I	15	R	Assessment of prolong overall survival or disease-free survival in patients with recurrent glioblastoma	NCT06757153	n/a
		TS-2021	I	30	R	Assessment of safety and efficacy in malignant recurrent gliomas	NCT06585527	n/a
	**Polio/** **rhinovirus**	PVSRIPO	II	121	ANR	Evaluation of safety & efficacy in recurrent glioblastoma	NCT02986178	(108)
Cytokine therapy		L19TNF +/- TMZ	I/II	226	R	Exploring the safety profile and establish a recommended dose in newly diagnosed glioblastoma	NCT04443010	n/a
		L19TNF + Lomustine	I/II	142	R	Safety & efficacy evaluation in patients with glioblastoma in first progression	NCT04573192	(109)
		Tamferon™	I/IIa	27	R	Evaluation of safety and efficacy in patients with GBM and unmethylated MGMT gene promoter	NCT03866109	(110)
		Tocilizumab + Atezolizumab + RT	II	59	ANR	Assessment of efficacy & MTD	NCT04729959	(111)
Other targeted immunomodulatory therapies including monoclonal antibodies		Autologous TLI + Pembrolizumab	I/II	85	R	Assessment of safety & efficacy	NCT06640582	n/a
		IGV-001 + RT + TMZ	IIb	93	ANR	Assessment of safety and efficacy in newly diagnosed glioblastoma patients that underwent surgical resection	NCT04485949	(112)
		Daratumumab (Darzalex) + RT + TMZ	I/II	16	ANR	Assessment of enhanced antitumor efficacy in glioblastoma	NCT04922723	n/a
		D2C7-IT + 2141-V11	I	46	R	Assessment of safety and efficacy in resected recurrent glioblastoma	NCT06455605	n/a
		D2C7-IT & 2141-V11	I/II	50	R	Determination of safety and efficacy in newly diagnosed glioblastoma	NCT05734560	n/a
		Indoximod +/- TMZ +/- RT +/- Cycliphophamide +/- Etoposide +/- Lomustine	II	140	R	Assessment of efficacy in pediatric glioblastoma	NCT04049669	(113)
		N-803 + PD-L1 t-haNK + Bevacizumab	II	20	R	Evaluation of safety and efficacy in progressive or recurrent glioblastoma	NCT06061809	n/a
		NGM707 (anti-ILT2 and ILT4) +/- Pembrolizumab	I/II	179	ANR	Dose escalation/expansion study in advanced or metastatic glioblastoma	NCT04913337	(114)

DC, Dendritic cell; SOC, Standard of care; CMV, cytomegalovirus; WT1, Wilm’s tumor 1, IDH, Isocitrate dehydrogenase; MGMT, O^6^-methylguanine-DNA methyltransferase; hTERT, human telomerase reverse transcriptase; TMZ, Temozolomide; pp65, lower matrix protein 65; PD-L1, programmed death-ligand 1; t-haNK, targeting high affinity Natural Killer cells; MTD, maximum tolerated dose; oHSV, oncolytic herpes simplex virus; oAds, oncolytic adenovirus; IDO, indoleamine 2,3-dioxygenase; EphA2, Ephrin type-A receptor 2; RT, Radiotherapy; NeoAg, Neoantigen; TERT, telomerase reverse transcriptase; PTPRZ1, Protein tyrosine phosphatase receptor type Z1; fl, full length; LPs, lipid particles; MTA, Mutated-derived tumor antigen; poly-ICLC, Polyinosinic-Polycytidylic acid; pINF, plasmid interferon; GSCs, glioblastoma stem cells; TTFs, Tumor-treating fields; TNF, Tumor necrosis factor; ILT2/4, Immunoglobulin-like transcript 2/4; n/a, not applicable.

### 4.1 Immune checkpoint inhibitors

Immune checkpoints are the gatekeepers of the immune system, crucial for preventing autoimmunity under normal physiological conditions and protecting tissues from damage following response of the immune system to pathogens (115). In cancer, the expression of immune-checkpoint proteins is dysregulated and therefore confers immune resistance. Several inhibitory immunoreceptors, referred to as “immune checkpoints”, have been identified and studied in cancer, including, amongst others, programmed death ligand 1 (PD-1), cytotoxic T-lymphocyte associated protein 4 (CTLA-4), lymphocyte activation gene 3 protein (LAG3), T cell immunoglobulin and mucin domain-containing protein 3 (TIM3), and T-cell immunoreceptor with immunoglobulin G1 (Ig1) and immunoreceptor tyrosine-based inhibitory motif (ITIM) domains (TIGIT) (116). Blocking their activity with ICIs has revolutionized cancer therapy over the past few years and has been successful for several cancer types (18, 19). Although initial trials with ICIs in glioblastoma revealed disappointing results (20), glioblastoma tumors are still considered a promising candidate for immunotherapy and is therefore being investigated in several clinical trials (**Tables 1**-**3**, **Figures 2**, **3**).

#### 4.1.1 Anti-PD-1/PD-L1

PD-1 receptor, expressed on T- and other immune cells, is a dominant negative regulator of T-cell response, when activated by its ligand, PD-L1, which is expressed on tumor cells (117). Anti-PD-1/PDL-1 therapy has been approved for the treatment of several cancer types, including metastatic melanoma, non-small cell lung cancer (NSCLC), head and neck cancers, urothelial carcinoma and others (118). More specifically, there are 6 FDA-approved PD-1/PD-L1 inhibitors, including the PD-1 inhibitors Pembrolizumab (Keytruda), Nivolumab (Opdivo) and Cemiplimab (Libtayo) and the PD-L1 inhibitor Atezolizumab (Tecentriq). PD-1/PDL-1 therapy has been explored in several clinical studies in glioblastoma, and although it is safe, it did not prolong OS (18). Currently, there are no FDA-approved anti-PD-1/PD-L1 inhibitors for glioblastoma (119), but the PD-1 inhibitors Pembrolizumab, Nivolumab, Cemiplimab, Retifanlimab by Zynyz (approved under FDA’s Accelerated Approval Program for Merkel cell carcinoma), and Balstilimab (currently in Phase II clinical trials for several cancers), as well as the PD-L1 inhibitor, Atezolizumab, are being tested in clinical trials for safety, tolerability, feasibility and efficacy in newly diagnosed, recurrent and progressive glioblastoma, both as monotherapy and as part of a therapeutic regime (**Table 1**).

#### 4.1.2 Anti-CTLA-4

CTLA-4 belongs to the superfamily of CD28–B7 immunoglobulins and it shares its two ligands (B7.1, B7.2) with its co-stimulatory counterpart CD28, and together these molecules are functioning at the tip of the immunological cascade (120). In glioblastoma, CTLA-4 competes with CD28 for binding to costimulatory molecules (CD80 and CD86) on APCs, thereby inhibiting the activation of T cells. Anti-CTLA-4 as a single form of therapy or in combination with other ICIs, enhances endogenous immune responses to immunogenic tumors. Ipilimumab (MDX-010 and Yervoy^®^) is a humanized monoclonal CTLA-4 antibody that has been approved by the FDA as monotherapy in anti-PD-1 refractory cases or in combination with nivolumab as a first-line treatment of advanced melanoma (121). In glioblastoma clinical trials, ipilimumab is administered in combination with nivolumab (NCT06097975, **Table 1**). Botensilimab (AGEN1181), is an Fc-enhanced anti-CTLA-4 antibody, which is considered one of the most advanced-next generation ICIs currently in clinical trials, due to its novel FcyR-dependent mechanism to promote superior priming and activation of T cells (121). In glioblastoma, Botensilimab is being tested in a single clinical trial in combination with Balstilimab and chemotherapy, given through a BBB sonication device (NCT05864534, **Table 1**).

#### 4.1.3 Anti-TIM3

TIM-3 is an immuno-myeloid cell surface marker specific to IFN-γ producing CD4+ and CD8+ T cells, expressed on multiple immune cells and leukemic stem cells (122). In cancer, elevated TIM-3 expression is associated with poor outcome, therefore TIM-3 has become an attractive candidate for immunotherapy. Sabatolimab (MBG453), is a novel high-affinity, humanized, IgG4 antibody targeting the TIM-3 receptor currently under clinical development by Novartis for the treatment of both solid tumors and hematological malignancies (123). Sabatolimab, is tested for safety in a Phase I clinical trial of recurrent glioblastoma, in which is given in combination with spartalizumab (anti-PD-1) (NCT03961971, **Table 1**).

#### 4.1.4 Anti-TIGIT

TIGIT belongs to the Ig superfamily, and it is expressed on activated CD4+ and CD8+ T cells, as well as NK cells (124–126). TIGIT interacts with its ligand, CD155, which is expressed mostly on DCs and macrophages, and upon TIGIT/CD155 interaction, immune responses are negatively regulated. Specifically, T-cell receptor expression is reduced, resulting in impairment of the function of CD8+ and NK cells, resulting in immunosuppression. Currently, Domvanalimab, an investigational inhibitor, is being evaluated in clinical trials in combination Zimberelimab (anti-PD-1) in recurrent glioblastoma (NCT04656535, **Table 1**).

Despite extensive research, there is still no ICI approved for the treatment of glioblastoma, since no significant improvement in OS has been observed during clinical trials so far. Despite the disappointing early clinical trial results, the use of ICIs for the treatment of glioblastoma remains under ongoing clinical investigation, aiming to reverse the glioblastoma immunosuppressive TME which is mainly attributed to the upregulation of several immune checkpoint molecules, such as PD-1, PD-L1, CTLA-4, LAG-3, TIM-3 and TIGIT. A variety of ICIs are currently being investigated in clinical trials in glioblastoma (early, aggressive or recurrent), for their safety and effectiveness, often in combination with other therapies to overcome the limitations of monotherapy and improve therapeutic outcomes. The use of novel delivery systems in several trials, like the Optune^®^ device, suggests a commitment in overcoming delivery limitations especially the BBB. Furthermore, combining ICIs with personalized neoantigen vaccines aims to enhance T-cell activation and specificity. In addition, targeting myeloid cells aims to enhance antigen presentation. Importantly, discovery of new biomarkers to predict response to ICI therapy is essential in GMB; for example, in other types of cancer, patients with low PDL-1 levels also benefit from anti-PD-1 therapy, suggesting other mechanisms are involved in their action (127). The lack of reliable biomarkers for the use of ICIs in GMB is discussed in recent reviews (128, 129). The future of ICIs in glioblastoma, depends on the delivery systems, the drug combination strategies, the tackling of the cold glioblastoma immune microenvironment, and the identification of personalized biomarkers based on molecular signatures or immune profiles that could help tailor ICI therapies to responsive subpopulations of patients. Another important factor is optimizing the timing of ICI administration. For instance, recent findings suggest that administering combination ICI in the neoadjuvant setting can stimulate the infiltration, activation, and proliferation of tumor-specific T cells in patients with newly diagnosed glioblastoma (130). In fact, a clinical trial, based on this, is already ongoing (NCT06816927).

### 4.2 T-cell based therapies

#### 4.2.1 CAR T-cells

CAR-T cell therapy is at the forefront of T-cell based therapies, providing a powerful tool for cancer treatment (131–133). It involves the inducible expression of a chimeric antigen receptor (CAR), engineered to target a specific antigen of interest on autologous T cells, hence bypassing the need for antigen presentation by Major Histocompatibility Complex (MHC) otherwise required for the activation of endogenous T cells (131–134). Since 2017, six CAR-T cell products have been approved for the treatment of several cancers, including acute lymphoblastic leukemia (ALL), diffuse large B-cell lymphoma (DLBCL), refractory follicular lymphoma (FL), recurrent or refractory mantle cell lymphoma (MCL), and relapsed or refractory multiple myeloma (133). Currently there are no FDA-approved CAR-T cell therapies for glioblastoma, but several clinical trials (mostly in Phase I) are evaluating CAR-T cell therapy in glioblastoma (**Table 2**; **Figure 2B**), including one that is combining CAR-T cell therapy with ICIs (NCT04003649). The glioblastoma antigens currently targeted in the clinic, often in combination, are Cluster of Differentiation proteins (CD133, CD44 & CD70), IL receptors (IL-13Ra2), EGFR and EGFRvIII, ephrin receptor A2 (EphA2), human epidermal growth factor receptor 2 (HER2) and B7-H3 (**Table 2**).

IL-7 is a hematopoietic cytokine that promotes the activation, differentiation and homeostasis of naïve T-cells, as well as the survival, expansion and proliferation of memory T-cells (135). It has been shown that engineered T-cells constitutively expressing the IL-7 receptor alpha (IL-7Rα) have great antitumor efficacies in both breast cancer (136) and glioblastoma models (137), therefore, IL-7 and its receptor are great candidates for immunotherapy. For the clinical trial NCT05577091 (**Table 2**), autologous T-cells have been genetically modified to express a CAR targeting CD133 and CD44, and a truncated form of the IL-7Rα. These CAR-T cells are believed to have immunostimulating and anti-neoplastic activities since they target CD133 and CD44, two markers of GSCs, associated with the proliferative or invasive state of glioblastoma cells (138). Also, the IL-7Rα-Tris-CAR-T cells induce selective toxicity to tumor cells, and the IL-7/IL-7Ra-mediated signaling promotes the proliferation and survival of T cells.

IL-8 is another important chemokine, that coupled with its receptor, IL-8R, play a role in tumor invasion, proliferation, survival and angiogenesis, as well as in the promotion of the malignant properties of the glioblastoma stem cells (139–142). CD70, is an antigen that is overexpressed in glioblastoma and is associated with poor survival (143). It was also hypothesized that it correlates with a mesenchymal phenotype and immunosuppression via recruitment of macrophages and CD8+ T-cell death. This information on IL-8 and CD70, let to the generation of CD70-targeting CAR with a modified IL-8 receptor (called 8R-70CAR) that let to complete tumor regression of advance cancers in pre-clinical studies, including glioblastoma (144). (NCT05353530 & NCT06946680, **Table 2**).

IL-13Rα2 is a high affinity membrane receptor that is overexpressed in glioblastoma, and is associated with poor outcome, mesenchymal gene profile, immunity, and the tumor microenvironment, which make it an important therapeutic target (145, 146). First-generation of IL-13Rα2-targeted CAR-T cells showed evidence of antitumor efficacy, but limited persistence of T-cells (147), therefore new approaches were explored in the design and production of IL-13Rα2-targeted CAR-T cells. Brown et al. (2018) (148), designed a second-generation of IL-13Rα2-targeted CAR, by engineering memory-enriched T cells to express IL-13Rα2 and 41BB-constimulatory CAR, a member of the tumor necrosis factor (TNF) receptor superfamily that enhances CAR-T survival and persistence (149, 150). Furthermore, the engineered T-cells expressed a truncated form of CD19, a pan B-cell marker (151) that has been targeted for the treatment of several hematological malignancies (152), and was shown to be highly expressed in brain and endothelial cells causing increased BBB permeability and neurotoxicity after targeted CAR-T immunotherapy (153, 154). These second-generation CAR-T cells not only improved anti-tumor activity but also T-cell persistence. Furthermore, it was shown that when delivered intracranially, they had a greater anti-tumor effect compared to intravenous delivery in orthotopic glioblastoma mouse models. This study, led to the protocol design for the NCT02208362 trial (**Table 2**), in which multiple intracranial infusions of these CAR-T cells, were not only proven to be safe, but also let to the regression of glioblastoma, increased levels of immune cells and cytokines, and persistence of response for up to 7.5 months after treatment initiation (155). Results from this trial, demonstrated that locoregional therapy with IL-13Rα2-targeted CAR-T is safe with promising clinical activity in a subpopulation of patients (88). Later, Chang Xu et al. (2022) (156), developed the first humanized third-generation CAR-targeting IL-13Rα2 that showed great anti-tumor efficacy and reduced expression of immunosuppressive cytokines such as IL-6 (88, 157) Like second and third generation IL-13Rα2-targeted CAR-T cells, HER2-specific, 41BB-costimulatory CAR with a truncated CD19 have been engineered and are being assessed in recurrent or non-responsive glioblastoma (NCT03389230, **Table 2**). HER2 is another important target, since it was found to be overexpressed in glioblastoma and is associated with poor prognosis (158).

EGFRs are transmembrane receptors, part of the ErbB family of receptor tyrosine kinases (RTKs), activated by several ligands (e.g. TGFα) binding to the extracellular domain (ECD) (159). Ligand-receptor formation of homo- or hetero-dimers, leads to the activation of several downstream pathways (e.g. MAPK, STAT3 and PI3K) that regulate cell survival, proliferation, angiogenesis and migration. When EGFRs are overexpressed or mutated, they stay constitutively active, which leads to uncontrolled cell proliferation and therefore tumor progression. EGFR is overexpressed in ~60% of primary glioblastoma and 10% of secondary glioblastomas (160). Furthermore, several mutants have been found in glioblastoma, with the most common being EGRvIII (159, 160). Overexpression and mutations in EGFR lead to a more aggressive glioblastoma phenotype and increased tumor heterogeneity, therefore several strategies have been developed to target EGFR (**Table 2**) (87, 89, 161, 162) B7-H3 (also known as CD276) is a type I transmembrane protein, that expressed in >70% of glioblastoma patients (163, 164), and is associated with progression, metastasis, poor outcome and immune evasion (165), as it is an immune checkpoint molecule expressed on antigen-presenting cells. The antitumor efficacy of B7-H3 CAR-T cells in glioblastoma *in vitro and in vivo* models was first reported in Xing Tang et al. (2019) (166). Several clinical trials, currently in recruiting phase, in refractory or recurrent glioblastoma, are evaluating the safety, feasibility, dose and efficacy of B7-H3 CAR-T cells (NCT05835687, NCT06482905, NCT05366179 & NCT05474378 **Table 2**). In NCT05241392 (**Table 2**), the preliminary data were recently published and showed that the use of B7-H3 CAR-T cells is safe and tolerated and holds great promise toward improving patients’ overall survival (167). Regarding the pharmacokinetic profile of B7-H3 CAR-T cells, a study reported that local delivery led to cerebrospinal fluid (CSF) persistence and localized immune activation rather than systemic effects (168). It is worth noting, that in a recent pre-clinical study, B7-H3 CAR-T cells consisting of IL-7Rα, have been shown to suppress tumor growth and prolong overall survival in glioblastoma mouse models (169), suggesting the potential implication of B7-H3-IL-7Rα CAR-T cells in the clinic.

Finally, chlorotoxin (CLTX) is a small 36-amino-acid peptide purified from the venom of the scorpion L*eiurus quinquestriatus* (170). CLTX was initially characterized based on its inhibition of glioma-specific chloride ion channels (GCC), however recent studies identified MMP-2 as the principal receptor for CLTX on the surface of glioblastoma cells. As mentioned above, MMP-2 expression is increased in glioblastoma TME, contributing significantly to the tumor invasiveness. CLTX has demonstrated specific and selective binding to membrane-bound MMP-2 and minimal binding to normal brain tissue, therefore CAR-T cells engineered to incorporate CTLX as an antigen recognition domain are considered a promising approach for MMP-2 positive glioblastoma treatment, redirecting cytotoxic T cells towards glioblastoma cells (171).

#### 4.2.2 (γδ)T-cells

(γδ)T-cells comprise a unique type of innate immune T cells that express a γδ T cell receptor (TCR), and are found abundant in several tissues, including lymphocytes that infiltrate solid tumors (172, 173). They can directly kill tumor cells through (a) cytokine-mediated cytotoxicity (e.g. TNFα and IFNγ), (b) perforin (PFN) & granzyme (GzmB) and FasL & TRAILR mediated target cell apoptosis, (c) antibody-dependent cell-mediated cytotoxicity, and (d) antigen processing and presentation. They can also have indirect anti-tumor effects through interaction with immune cells (e.g. NK cells and αβ T-cells). In addition, they have no autologous limitations, can be derived from healthy donors, and can be easily expanded. These properties make them a powerful tool in immunotherapy. The first therapeutic mechanism involves the selective amplification of (γδ)T-cells *in vivo* using antibodies or bisphosphonate antigens. In the second mechanism, which is adoptive cell therapy, tumors are treated with allogeneic (γδ)T-cells (natural or genetically engineered) or (γδ)T-cells that have been expanded *in vitro*. The first-in-human Phase I clinical trial (NCT04165941, **Table 2**) involves the intracranial infusion of TMZ-resistant (γδ)T-cells in newly diagnosed glioblastoma patients. During TMZ treatment, the natural killer group 2, member D (NKG2D) receptor ligands (NDG2DL), the master activators of NK cells (174), are upregulated, but this immune response is impaired due to lymphodepletion (172, 175). Therefore, genetic modification of (γδ)T-cells, naturally expressing the NKG2D receptor, were genetically modified and expanded *ex vivo* with an MGMT-expressing lentivector that provided resistance to TMZ, allowing therefore the simultaneous infusion of (γδ)T-cell with chemotherapy, and targeting therefore the tumor when NKG2DL are maximally expressed (**Figure 2C**). Modifying the cells to be resistant to TMZ is crucial, as they need to be able to resist the cytotoxic effects of TMZ and be able to survive and function on the presence of chemotherapy. In another trial, gene modified allogeneic or autologous (γδ)T-Cells (DeltEx), again conferring TMZ resistance, are evaluated for safety, tolerance and ability to delay recurrence in newly or recurrent glioblastoma (NCT05664243, **Table 2**; **Figure 2C**).

The use of CAR-T cells in glioblastoma is based on targeting the tumor-specific or tumor-enriched antigens like IL-13Rα2, EGFR/EGFRvIII, HER2, CD70, B7-H3, and CD133/CD44. Apart from antigen-specificity, the use of innovative constructs in active clinical trials, such as next-generation CAR-T that integrate co-stimulatory domains, can improve T-cell persistence, activation, and tumor selectivity. In some trials, locoregional delivery of CAR-T cells through intracranial administration aims to enhance the efficacy and reduce systemic toxicity, although is more invasive and complex. Since glioblastoma is characterized by profound heterogeneity that limits the durability of response due to downregulation of target antigens, CAR-T cells that target multiple antigens have been developed that aim to reduce escape via antigen heterogeneity. (γδ)T-cells use a different strategy from CAR-T, exploiting innate-like immune response. Based on their recognition of ligands independently of MHC, they are ideal for glioblastoma that is immune-evasive. Another advantage is that they are multifunctional, since use different mechanisms for killing tumor cells. On the other hand, the safety and efficacy of (γδ)T-cells is still not well established and they also require complex genetic engineering. Although highly innovative, both CAR-T and (γδ)T-cells need to overcome antigen escape, and the suppressive microenvironment. Moving forward, further clinical exploration is needed, and their success will depend on effectively reshaping the TME.

### 4.3 Vaccines

As recently reviewed, cancer vaccines are another promising form of immunotherapy in glioblastoma (176). Briefly, anti-cancer vaccines aim to provoke an immune response within the body against tumor-specific antigen(s) and are generally composed of the antigen (neoantigen, tumor-associated antigen or pathogen derived-antigen) and the platform/carrier type (DC vaccine, peptide vaccine, nucleic acid vaccine or viral vector vaccine). Several vaccines are in active clinical trials in glioblastoma (**Table 2**, **Figure 3A**).

#### 4.3.1 Peptide vaccines

Peptide vaccines are composed of 8–30 amino acid including tumor-specific or tumor-associated antigens that elicit an anti-tumor T cell response (177, 178). As they are not highly immunogenic, they can be combined with other forms of immunotherapy or chemoradiation. They can be individualized, single-targeted or multi-targeted. One target of peptide vaccines is the survivin protein; a member of the inhibitor of apoptosis (IAPs) family, that is overexpressed in glioblastoma and is associated with poor prognosis (179). The survivin peptide vaccine, SurVaxM, is currently being evaluated in two clinical trials in combination with TMZ (NCT02455557 & NCT05163080, **Table 3**). Preliminary results showed that SurVaxM is safe and well-tolerated and its combination with TMZ is very promising (96). pp65 and survivin are also being targeted together with EphA2 through a multi-peptide vaccine (ETAPA I) in HLA-A*0201 positive patients with a newly diagnosed, unmethylated, and untreated glioblastoma (NCT05283109, **Table 3**). pp65 is overexpressed in high grade gliomas and medulloblastomas, but not in adjacent brain, and plays a significant role in glioblastoma progression. When tested in children and young adults, it was proven to be well-tolerated and promoted antigen-specific immune responses (180). Other targets include Telomerase Reverse Transcriptase (TERT), Protein tyrosine phosphatase receptor type Z1 (PTPRZ1) and Toll-like receptors (TLRs), that are being targeted in combination in NCT06622434 (**Table 3**). PTPRZ1 is a clinically relevant antibody in glioblastoma associated with stemness (181), and telomerase (TRT) is a major oncogene, whose promoter is mutated in approximately 80% of glioblastoma patients and is associated with tumor progression (96, 182, 183). TLRs are ubiquitously expressed receptors that recognize pathogens and lie at the first line of defense in the innate immune system (184). In several tumor types, upon ligand recognition, TLRs activate downstream intracellular signaling pathways either supporting or suppressing tumor growth, thus they are a great candidate for immunotherapy. Finally, several autologous or allogeneic multipartite vaccines, designed to induce a variety of neoantigen-specific immune responses, are tested in clinical trials in combination with other therapies, such as ICIs and nucleic acid vaccines (NCT03223103 & NCT02287428, **Table 3**).

#### 4.3.2 DC vaccines

DC are the most superior APC cells of the immune system, thus playing a vital role in presenting antigens in the lymph nodes eliciting T-cell priming and distant anti-tumor response (185, 186). DC vaccines are generated by culturing hematopoietic progenitor cells or monocytes *ex vivo* in the presentation of a cytokine cocktail to induce their maturation. Following maturation, DCs are loaded into tumor antigens and subsequently injected into patients. Not only they can achieve priming of CD4+ T cells by peptide-MHCII complex, but also, they elicit CD8+ T-cell antitumor responses. Several clinical trials are underway in glioblastoma investigating the safety and efficacy of DC vaccines in newly diagnosed or recurrent glioblastoma (**Table 3**). Some are loaded with autologous tumor lysates (NCT03395587, NCT04523688 & NCT04801147, **Table 3**), and even “double-loaded” (NCT06805305), and others are loaded with multiple tumor neoantigens (NCT04968366 & NCT06253234, **Table 3**). They are administered either alone or in combination with chemoradiation or ICIs. Finally, other DC vaccines are loaded with mRNAs for specific proteins, including Survivin/hTERT derived from autologous GSCs (NCT03548571, **Table 3**), the tumor-associated antigen Wilm’s tumor 1 (WT1) (NCT02649582, **Table 3**) and the human CMV immunodominant protein pp65-LAMP (NCT03688178, **Table 3**).

#### 4.3.3 Nucleic acid-based vaccines

Other than the DC and peptide vaccines, a few other vaccines are being exploited in glioblastoma immunotherapy trials based on the delivery of nucleic acids (**Table 3**). Nucleic acid-based vaccines introduce a segment of DNA or RNA that encodes a specific or multiple tumor antigens, to elicit and immune response (176). The current vaccines clinical trials include DNA vaccines (NCT05698199, & NCT04015700), an mRNA vaccine (NCT05938387), and two vaccines that use lipid nanoparticle technology (187) to deliver RNA (NCT04573140 & NCT06389591). Apart from the mRNA vaccine that encodes several GBM peptides (188), all the others are targeting the immunogenic and viral antigens of CMV.

Cancer vaccines are a growing field in glioblastoma immunotherapy, and they hold a great promise, since they aim to evoke tumor-specific immune response through tumor associated or neoantigen presentation. In addition, they have shown great tolerability and minimal toxicity in early-phase trials (e.g. SurVaxM). Furthermore, they allow personalized and/or multi-antigen targeting to address the heterogeneity present in glioblastoma. Especially DC vaccines, that are loaded with autologous tumor lysates, offer T-cell priming, overcoming the limitations of peptide vaccines (priming of weak immune response). The efficacy of vaccines might be limited the high infiltration of Tregs, and antigenic heterogeneity, therefore combining vaccines with other immunotherapies (CAR-T cells, ICIs and oncolytic viruses) may further improve immunogenicity and efficacy. Future progress hinges on optimizing vaccine formulations for better delivery, refining antigen targets, and leveraging synergies with other immunotherapies.

### 4.4 Oncolytic viruses

The development, use and clinical relevance of oncolytic virus in cancer immunotherapy have been extensively reviewed elsewhere (189–192). Briefly, oncolytic viruses (Ovs) are genetically engineered viruses that selectively attack and lyse tumor cells without disrupting normal cells via different biological mechanisms. They are manipulated in such way to enhance tumor selectivity, promote replication competence, limit pathogenicity and increase immunogenic cell death (ICD). In glioblastomas, treatment with OVs is very suitable due to their alignment to the brain environment, the fact that they do not form distant metastases, and finally that they are fast growing tumor cells that attract virus replication. OVs are classified into two major groups: (1) replication-competent OVs that selectively replicate in cancer cells e.g. Herpes Simplex Virus (oHSV) and adenoviruses (oAds) and (2) replication-deficient viral vectors used as vehicles for other therapeutic genes e.g. polioviruses.

Most ongoing clinical trials in glioblastoma are using oHSVs (**Table 3**, **Figure 3B**) (193–197).

Apart from oHSVs, trials utilizing oAds are currently ongoing in glioblastoma (**Table 3**, **Figure 3B**). DNX-2401 oAds has two stable genetic changes the dsDNA adenovirus genome that allows the selective and efficient replication in current cells (197). When combined with pembrolizumab, DNX-2401 not only was proven safe, but it also induced durable cell death by direct oncolytic activity and immune response in high-grade glioma patients. The drug is now in Phase I clinical trials in patients with recurrent glioblastoma (NCT03896568, **Table 3**). However, DNX-2401 is not intended for systemic BBB penetration due to its viral nature and size (195). NSC-CRAd-S-pk7, is another very promising adenovirus developed against glioblastoma (198). It has several features including: (1) a survivin promoter for enhancing specific replication in tumor cells, since survivin is found to be overexpressed in glioblastoma, (2) a modified Ad5 protein through insertion of polylysine sequence (pk7), which binds to heparin sulfate proteoglycans also overexpressed in glioblastoma and (3) neural stem cells (NSCs) as its carrier, also contributing to selectivity. In Phase I trials, NSC-CRAd-S-pk7 was shown to be safe, improve OS and result in an increase of cytotoxic T-cells (105). A clinical trial against recurrent high-grade glioma is currently underway (NCT05139056, **Table 3**). NRG-103 is a novel gene therapy agent engineered to enhance the tumor-specific recognition and cytolytic activity of an oncolytic virus, while simultaneously eliciting a robust anti-tumor immune response. It leverages *in situ* transdifferentiation technology, incorporating multiple engineered mutations within the adenoviral genome. Notably, NRG-103 expresses two transcription factors capable of efficiently reprogramming residual glioblastoma cells into neuronal-like cells, thereby aiming to delay tumor recurrence and improve long-term survival. In preclinical models it exhibited significant anti-tumor activity, and its overall or disease-free survival is being evaluated in patients with recurrent glioblastoma (NCT06757153, **Table 3**). Lastly, the safety and efficacy of the oncolytic virus TS-2021, is evaluated in the clinical trial NCT06585527 (**Table 3**). TS-2021 is a third-generation oncolytic adenovirus that can efficiently target glioblastoma cells overexpressing Ki67 (proliferation marker) and TGF-β2 and can inhibit invasiveness through targeting of the MKK4/JNK/MMP3 pathway (199).

Finally, PVSRIPO is under clinical investigation for safety and efficacy in recurrent glioblastoma (NCT02986178, **Table 3**). PVSRIPO is a type 1 (Sabin) live-attenuated poliovirus vaccine that carries a heterologous internal ribosomal entry site (IRES) of human rhinovirus type 2 (HRV2) (200). Its synthesis allows selective expression in tumor cells after coupled with its receptor, CD155, expressed in several tumors including glioblastoma, exerting antitumor effects.

Ovs are an innovative class of immunotherapies that offer direct tumor cells lysis as well as indirect stimulation of anti-tumor immune response. They exhibit several advantages including their selective replication in tumor cells minimizing the damage to normal brain tissue (e.g. oHSV G207 and C134 are designed with deletions in neurovirulence genes to limit replication in healthy neural tissue while enhancing tumor-specific lysis) (201). They can induce ICD, reprogram the immunosuppressive microenvironment (e.g. CAN-3110) and improve T-cell activation (e.g. NSC-CRAd-S-pk7). On the other hand, glioblastoma heterogeneity and pre-existing immunity against viral vectors, can limit OV spread and propagation. Furthermore, and tumor cells can develop resistance through interferon signaling or downregulation of viral receptors (e.g., CD155 for PVSRIPO). Even though promising results have been demonstrated in early-phase trials, their benefit on PFS and OS must be demonstrated in Phase II/III trials. Ongoing advances in vector engineering, biomarker-guided patient selection, and combinations with other immunotherapies (e.g. with ICIs) are expected to shape the next generation of OV-based therapies.

### 4.5 Cytokine therapies

Another form of immunotherapy are the cytokine-based therapies that are very promising, as they can potentially alter the immunosuppressive microenvironment and trigger anti-tumor immunity (195). However, the systemic administration of therapeutic doses of pro-inflammatory cytokines can cause toxicity. To tackle this systemic toxicity, L19TNF was generated. Composed of TNF fused to the L19 antibody, L19TNF is a fully human antibody–cytokine fusion designed to selectively deliver TNF to tumors by binding a fibronectin epitope uniquely expressed in the tumor extracellular matrix, where it induces cell death, and at the same time the reduction of toxicity to healthy organs (202) (**Figure 3C**). Ongoing trials are investigating the safety, efficacy and recommended dose of intravenous administration of L19TNF in newly diagnosed (NCT04443010, **Table 3**) and recurrent glioblastoma (NCT04573192, **Table 3**), in combination with TMZ-based chemoradiation or lomustine accordingly. In another ongoing clinical trial in recurrent glioblastoma (NCT04729957, **Table 3**), the efficacy and MTD of tocilizumab in combination with atezolizumab and stereotactic RT is being investigated. Tocilizumab is an anti-IL-6 monoclonal antibody that reduces the body’s immune response and inflammation (**Figure 3C**). Therefore, it suppresses the inhibitory effect of immune cells surrounding glioblastoma and consequently allow atezolizumab, an anti-PD-L1 treatment, to activate the immune response against glioblastoma. Finally, Tamferon™, was designed to increase the production of IFNα to cause immune activation (203). More specific, CD34+ HSPCS are isolated from the patient and are transduced *ex-vivo* with a lentivirus expressing IFNα downstream a Tie2 promoter, so that IFNα expression is confined to Tie-2 expressing macrophages (TEMs) (**Figure 3C**). The safety and efficacy of Tamferon™, is evaluated in patients with MGMT-unmethylated glioblastoma (NCT03866109, **Table 3**).

Cytokine therapy is a highly promising but still evolving frontier. It has an immunomodulation potential, since it aims to reprogram the TME through cytokines such as TNF and IFNα, enabling a more effective anti-tumor response. Innovations like L19TNF and Tamferon™ help to overcome the systemic toxicity by directing cytokine action to the tumor site. Furthermore, the latter utilizes patient-specific, gene-modified immune cells, showing a shift towards personalized immunotherapy. Cytokine therapy is often combined with other therapies, including the standard of care, to address other resistance mechanisms. Like other immunotherapeutic approaches, immune evasion and tumor heterogeneity are still a concern. Furthermore, delivery is still an issue, especially since cytokines have a limited therapeutic window, and local overexpression can cause neurotoxicity or inflammation. The issues need to be addressed, and success in clinical trials remains to be proven.

### 4.6 Other targeted immunomodulatory therapies

Finally, a few other strategies that do not explicitly fall into the categories already discussed, have been developed to induce immune-specific responses and are under clinical evaluation in glioblastoma (**Table 3**). For example, Tumor-infiltrating lymphocytes (TILs) therapy, an innovative form of adoptive cell therapy that utilizes the patient’s own immune cells to target and destroy cancer cells, is given in combination with pembrolizumab in patients with advanced gliomas (NCT06640582, **Table 3**). In addition, IGV-001 that is being evaluated in NCT04485949 (**Table 3**), is a first-in-class autologous immunotherapeutic product from Goldspire™, that combines personalized whole tumor-derived cells with an insulin-like growth factor receptor 1 (IGF-1R) antisense oligonucleotide (IMV-001) in an implantable biodiffusion chambers (204). It has been reported that inhibiting IGF-1R can effectively suppress the growth of GBM cells directly or indirectly through suppression of cell proliferation or angiogenesis respectively (205–208). In addition, the N-803 from Anktiva, a modified IL-15-based fusion protein (IL-15Rα-Fc), functions as an immunostimulatory agent that drives the expansion and activation of NK cells and CD8^+^ T lymphocytes (209), is being evaluated in progressive or recurrent glioblastoma (NCT06061809). It is given in combination with PD-L1 targeting high-affinity NK (t-haNK) cells (210) and the anti-VEGF antibody, Bevacizumab. While N-803 generally demonstrate improved tolerability, long-term exposure may still carry immunological risks. Sustained stimulation of NK and CD8+ T cells can lead to chronic immune activation, increasing the risk of cytokine release syndrome (CRS), immune-mediated tissue damage and inflammatory toxicities such as fever, and hypotension. Finally, the drug NGM707 is being evaluated as monotherapy and in combination with pembrolizumab in advanced or metastatic glioblastoma in NCT04913337 (**Table 3**). NGM707, is a dual humanized monoclonal antibody that targets Immunoglobulin-like transcript (ILT)2 and ILT4 resulting in early efficacy and biomarker signals in advanced or metastatic solid tumors (113), probably through the generation of immune niche and immune-checkpoint blockage (211).

These novel immunomodulatory therapies for glioblastoma are conceptually sound and mechanistically diverse, representing a hopeful direction. TILs and the IGV-001 personalized vaccine provide tumor-specific cytotoxicity since the former are isolated from patient’s tumor and the latter combines patient-specific tumor cells with IGF-1R antisense oligonucleotide. Combination of TLIs with pembrolizumab maximizes activity by preventing T-cell exhaustion. Daratumumab and other monoclonal antibodies are designed against tumor-specific antigens, aiming to enhance DC priming and T-cell activation, boosting anti-tumor immunity. Indoximod and N-803 both enhance NK function, and finally NGM707 aims checkpoint inhibition beyond PD-1/PD-L1, as ILT2/ILT4 target innate immune checkpoints, potentially reviving exhausted myeloid cells and enhancing antigen presentation. However, despite their innovative approach, common hurdles persist including tumor heterogeneity leading to immune escape, impaired delivery due to the BBB and complex trial designs. Most evidence remains preclinical or Phase I, making long-term benefit speculative.

## 5 Conclusions & future prospectives

Glioblastoma remains among the most aggressive and therapeutically challenging malignancies due to its complex, heterogeneous, and profoundly immunosuppressive tumor microenvironment (TME). Despite the significant progress in cancer immunotherapy in some tumor types, mainly using ICIs and CAR-T cell therapies, these approaches have not yet demonstrated substantial clinical benefits in glioblastoma patients, primarily due to intrinsic resistance mechanisms facilitated by the glioblastoma TME. The etiology of this phenomenon is clearly multifactorial and is largely attributed to the highly immunosuppressive nature and heterogeneity of GBM tumors. Immunosuppression in the TME is mediated via a multitude of underlying mechanisms, including the secretion of immunosuppressive cytokines, the abundance of Tregs, MDSCs and GAMs in the TME, the insufficient infiltration and elimination of antigen-specific T cells, the sequestration of T-cells in the bone marrow leading to their dysfunction, T-cell exhaustion, antigen escape as well as upregulation of multiple immune checkpoint molecules. In addition, the low TMB present in the majority of GBM tumors leads to limited number of produced neoantigens, which are needed to elicit durable T-cell responses, and contributes to the limited efficacy of immunotherapy. Moreover, physiological barriers like the blood-brain barrier (BBB), along with hypoxic and acidic conditions within the TME, significantly hinder therapeutic efficacy and immune response.

This review provides a comprehensive description of immunotherapy clinical trials. However, interpretations based on these trials are limited due to mixed treatment regimens (monotherapy *vs*. combinations) and ethnic bias, as many cited studies were conducted in predominantly Japanese cohorts, reducing generalizability across broader populations. To enhance the success of glioblastoma immunotherapies, future strategies must involve a comprehensive and functional understanding of distinct components of the TME at the single-cell level, such as tumor profiling using spatial transcriptomics and proteomics as well as patient-specific neoantigen identification, that will enable personalized and precision targeting to improve immunotherapy efficacy and patient outcomes. In addition, advancing glioblastoma therapy requires overcoming simultaneously both the biological and physical barriers that influence the therapeutic efficacy, such as the immunosuppressive TME, ECM composition and BBB permeability. Therefore, technological advancements in BBB penetration and targeted drug delivery (e.g., advanced nanomedicine, ultrasound-mediated BBB disruption) hold substantial promise and should be further explored.

In addition, combining immunotherapeutic agents with strategies that modulate the non-cellular components of the TME, such as agents targeting hypoxia, acidosis, and ECM constituents, could also enhance therapeutic outcomes. Further exploration of combination therapies integrating ICIs, CAR-T cells, vaccines, and oncolytic viruses with standard therapies (chemotherapy, radiotherapy) and novel targeted treatments is critical. However, mitigating short- and long-term side-effects in patients, especially in combination treatments likely remains one of the major challenges that need to be addressed.

To advance the field of glioblastoma immunotherapy, future efforts should move beyond incremental improvements. One promising direction is the application of artificial intelligence (AI) and machine learning (ML) to optimize clinical trial design, enabling real-time patient stratification and prediction of therapeutic response based on multi-omics data. Subsequently, spatial mapping of the TME through spatial transcriptomics and multiplexed imaging can uncover regional immune niches and patterns of immune suppression within glioblastoma, guiding the localization of targeted therapies. Coupling these insights with biomarker-driven patient stratification could personalize immunotherapy approaches and increase clinical efficacy.

Finally, integrating novel biomarkers and robust pre-clinical models using state-of-the art humanized patient-derived xenograft models into clinical trial designs will facilitate better patient stratification, treatment personalization, and evaluation of immunotherapy efficacy. Future interdisciplinary research efforts must focus on refining patient selection criteria and developing multimodal therapies that target both the tumor and its immunosuppressive milieu. These will be crucial in overcoming existing barriers, emphasizing on exploiting glioblastoma-specific vulnerabilities to ultimately transform the treatment landscape for glioblastoma. Despite numerous challenges, immunotherapy remains one of the most promising treatment strategies for glioblastoma.
